# Molecular phylogeny of *Panicum* s. str. (Poaceae, Panicoideae, Paniceae) and insights into its biogeography and evolution

**DOI:** 10.1371/journal.pone.0191529

**Published:** 2018-02-21

**Authors:** Fernando Omar Zuloaga, Diego Leonel Salariato, Amalia Scataglini

**Affiliations:** Instituto de Botánica Darwinion, San Isidro, Buenos Aires, Argentina; Indiana University Bloomington, UNITED STATES

## Abstract

*Panicum* sensu stricto is a genus of grasses (Poaceae) with nearly, according to this study, 163 species distributed worldwide. This genus is included in the subtribe Panicinae together with *Louisiella*, the latter with 2 species. *Panicum* and subtribe Panicinae are characterized by including annual or perennial taxa with open and lax panicles, and spikelets with the lower glume reduced; all taxa also share a basic chromosome number of *x* = 9 and a Kranz leaf blade anatomy typical of the NAD-me subtype photosynthetic pathway. Nevertheless, the phylogenetic placements of many Panicum species, and the circumscription of the genus, remained untested. Therefore, phylogenetic analyses were conducted using sequence data from the *ndhF* plastid region, in an extensive worldwide sampling of *Panicum* and related genera, in order to infer evolutionary relationships and to provide a phylogenetic framework to review the classification of the genus. Diversification times, historical biogeography and evolutionary patterns of the life history (annual vs. perennial) in the subtribe and *Panicum* were also studied. Results obtained provide strong support for a monophyletic *Panicum* including 71 species and 7 sections, of which sections *Arthragrostis* and *Yakirra* are new in the genus; 7 new combinations are made here. Furthermore, 32 species traditionally assigned to *Panicum* were excluded from the genus, and discussed in other subtribes of Paniceae. Our study suggested that early diversification in subtribe Panicinae and *Panicum* occurred through the Early-Mid Miocene in the Neotropics, while the subsequent diversification of its sections mainly occurred in the Late Miocene-Pleistocene, involving multiple dispersals to all continents. Our analyses also showed that transition rates and changes between annual and perennial life history in *Panicum* were quite frequent, suggesting considerable lability of this trait. Changes of the life history, together with C_4_ photosynthesis, and the multiple dispersal events since the Mid Miocene, seem to have facilitated a widespread distribution of the genus. All these findings contribute to a better understanding of the systematics and evolution of *Panicum*.

## Introduction

Within flowering plants, including grasses, reproductive characters have traditionally formed the backbone of hierarchical classifications. This scheme in many cases conflicted with molecular phylogeny research, which produced a new classification system; [1–6] in grasses. This is particularly true for huge genera, such as *Panicum* L., which in its broad sense is non-monophyletic, as well as *Senecio* [7], *Acacia* [8–9]), and *Aster* [10–11]. In this regard, *Panicum* is still maintained as a polyphyletic genus by some authors [12–17]), while others, summary in [16–17], treated species of the genus in other tribes, subtribes, and genera of subfamily Panicoideae or established new taxa to accommodate these segregate species. *Panicum* L., as traditionally circumscribed, was one of the largest genera of the Poaceae [12], with nearly 450 species distributed worldwide and inhabiting habitats from sea level to approximately 2500 m [18]. The main character placing species in the genus was the spikelet structure, with a lower glume present, usually shorter than the upper glume and lower lemma, the latter subequal, a lower flower present or absent, the upper anthecium indurate and abaxially convex, and a caryopsis with a punctiform to oblong hilum; nevertheless these characters also appear in other members of tribes Paniceae and Paspaleae. *Panicum* s. l. also exhibits differences in inflorescence types, developmental patterns of spikelets, including nervation of the glumes, and texture and ornamentation of the upper anthecium. Furthermore, physiological, anatomical and cytological diversity is present in *Panicum* s.l.: all known photosynthetic types found in grasses, occur in the genus, with many non-Kranz species gathered together with all Kranz variants of C_4_ physiology, i.e., NADP-me, NAD-me and PEP-ck subtypes; also, some species are intermediate between the C_3_ and C_4_ pathways [19–21]. In addition, two basic chromosome numbers were reported for the genus, with some species *x* = 9 and others *x* = 10.

Phylogenetic studies, based on morphological and molecular characters, have demonstrated that *Panicum* in its traditional sense [13–15] is not monophyletic [22–29] and that it should be restricted to a set of species all using the C_4_ NAD-me photosynthetic subtype. These studies implied several changes during the shift from schemes based exclusively on morphological data [12, 30] to those based on molecular data, with new delimitations within the Panicoideae. In the new classification scheme proposed by [16], and [5–6], species traditionally grouped in *Panicum s*.*l*. were included under three different subtribes of tribe Paspaleae (Arthropogoninae, Paspalinae, and Otachyriinae) and five subtribes of Paniceae: Boivinellinae, Cenchrinae, Dichanthelliinae, Melinidinae, and Panicinae, [31–51], Table 1.

**Table 1:** Placement of taxa segregated from *Panicum* in tribes and subtribes of supertribes Andropogonodae and Panicodae.

**Supertribe Andropogonodae**			
	**Tribe Paspaleae**		
		**Subtribe Paspalinae**	
		*Aakia* Grande Allende	[31]
		*Hopia* Zuloaga & Morrone	[32]
		*Ocellochloa* Zuloaga & Morrone	[33]
		*Osvaldoa* Grande Allende	[31]
		*Renvoizea* Zuloaga & Morrone	[34]
		**Subtribe Otachyriinae**	
		*Hymenachne* P. Beauv.	[21, 26]
		*Steinchisma* Raf.	[21, 35]
		*Rugoloa* Zuloaga	[21]
		**Subtribe Arthropogoninae**	
		*Apochloa* Zuloaga & Morrone	[34]
		*Canastra* Morrone, Zuloaga, Davidse & Filg.	[36]
		*Coleataenia* Griseb.	[37–38]
		*Cyphonanthus* Zuloaga & Morrone	[39]
		*Homolepis* Chase	[40]
		*Stephostachys* Zuloaga & Morrone	[41]
		*Tatiany* Zuloaga & Soderstr.	[40]
**Supertribe Panicodae**			
	**Tribe Paniceae**		
		“**Incertae sedis”**	
		*Homopholis* C.E. Hubb.	[42]
		*Kellochloa* Lizarazu, M.V. Nicola & Scataglini	[17]
		*Trichanthecium* Zuloaga & Morrone	[43]
		*Walwhalleya* Wills & J.J. Bruhl	[42]
		**Subtribe Dichantheliinae**	
		*Adenochloa* Zuloaga	[44]
		*Dichanthelium* (Hitchc. & Chase) Gould	[26]
		**Subtribe Boivinellinae**	
		*Morronea* Zuloaga & Scataglini	[45]
		*Parodiophyllochloa* Zuloaga & Morrone	[46]
		**Subtribe Cenchrinae**	
		*Whiteochla* C.E. Hubb.	[47]
		*Zuloagaea* Bess	[48]
		**Subtribe Melinidinae**	
		*Megathyrsus* (Pilg.) B.K. Simon & S.L. Jacobs	[49]
		**Subtribe Panicinae**	
		*Louisiella* C.E. Hubb. & Léonard	[50–51]

Subtribe Panicinae includes ca. 165 species, distributed worldwide, of *Panicum* s. str., and two American and African species of its sister genus *Louisiella* C.E. Hubb. & J. Léonard. Although over the last decade several grass phylogenies have been published for Panicoideae [1, 3, 23–26, 29, 52–55], species of *Panicum* s. str. were underrepresented; consequently, a study including a comprehensive sampling of the genus and the small genera related to *Panicum*, i.e., *Louisiella*, *Arthragrostis* Lazarides, *Whiteochloa* C.E. Hubb., and *Yakirra* Lazarides & R.D. Webster, is still needed. [5–6, 29, 53].

The aims of this study are to reconstruct the molecular phylogeny of subtribe Panicinae and *Panicum* s. str., using sequence data from the *ndhF* plastid region with an extensive sampling of *Panicum* and related genera, in order to test whether the current classification agrees with the phylogenetic history of the group, and to identify robust clades within the genus. Additionally, we also explore the divergence times for the subtribe and its members, the biogeographical events occurring over its diversification, and the evolutionary patterns exhibited by the life history (annual vs. perennial). Results obtained here are used to propose a new subgeneric classification for *Panicum*, and to elucidate different evolutionary insights from its diversification.

## Materials and methods

### Taxon sampling and DNA sequencing

In this study, we inferred a *ndhF* phylogeny because this marker has provided a robust and strong phylogeny of the Panicoideae and it has proven to be a useful tool to resolve different phylogenetic lineages of plants ([25–26, 29] and several other treatments summarized in Table 1), confirmed by phylogenies based in other genes [1, 3, 23–24, 28, 52, 54–55]. It is important to mention, however, that single-locus phylogenies (gene tree) can be discordant with the species tree, due to different processes such as lineage sorting, introgression, gene duplication, and strong positive selection. Additional multilocus analyses are needed to confirm results obtained here. Nevertheless, our analyses represent the first study to include an extensive sampling of *Panicum*. The *ndhF* matrix analyzed here consisted of 214 sequences, 70 of which were generated for this study to maximize the representation of *Panicum* species and allied genera (57 *Panicum*, *3 Yakirra*, 6 *Whiteochloa* and 4 *Arthragrostis*). The remaining 134 sequences were selected from Genbank based on the Panicoid matrix from [26], with the addition of *Panicum* and related species from [43, 50, 55–58]. In case of potentially uncertain or unexpected positions, two or more vouchers per species were analyzed [i.e. *P*. *laetum* Kunth, *Whiteochloa capillipes* (Benth.) Lazarides]. Information on specimen vouchers for the new sequences obtained and Genbank accessions for all species analyzed are provided in S1 Appendix.

Total genomic DNA was extracted from silica-dried leaves (7 taxa) and from herbarium specimens (63 taxa). DNA of silica samples was extracted with a CTAB protocol [59], while with herbarium material, the DNeasy Plant Mini Kit (Qiagen, Hilden, Germany) was used. The complete *ndhF* gene (ca. 2100 bp) was amplified using primers specified by [26, 60]. For silica-dried samples, three pairs of primers were used (5F-972R, 972F-1666R and 1666F-3R). For herbarium samples, five smaller fragments were amplified using the pairs of primers 5F-536R, 536F-972R, 972F-1666R, 1666F-1821R and 1821F-3R. PCR reactions were performed in 25 ul of final volume with 50–100 ng of template DNA, 0.2 uM of each primer, 25 uM of dNTP, 5 mM MgCl2, and 0.3 units of Taq polymerase provided by Invitrogen Life Technologies. PCR was carried out using the following parameters: one cycle of 94°C for 5 min, 39 cycles of 94°C for 30 s, 48°C for 1 min, and 72°C for 1 min 30 s, and a final extension cycle of 72°C for 10 min. For the species that failed this protocol, primer concentrations were varied. In addition, a variety of PCR additives and enhancing agents (bovine serum albumin, dimethyl sulfoxide) have been used to increase the yield, specificity and consistency of PCRs of herbarium samples. PCR products were run out on a 1% TBE agarose gel stained with SYBR Safe DNA gel stain (Invitrogen) and visualized in a blue-light transilluminator. Automated sequencing was performed by Macrogen, Inc.

Alignment was manually performed using BioEdit ver. 5.0.9 [60]. The aligned matrix is available online from the Dryad Digital Repository: doi:10.5061/dryad.286gn

### Phylogenetic analyses and molecular dating

First, the best-fitting codon partition scheme and model of sequence evolution for the *ndhF* dataset were determined using the Bayesian Information Criterion (BIC) in PartitionFinder 2.1.1 [61]. Three partition schemes corresponding with the codon positions were selected: 1^st^ pos. (TVM+I+G), 2^nd^ pos. (TRN+I+G), and 3^rd^ pos. (TVM+I+G). Maximum likelihood (ML) analyses were conducted in RAxML 8.2.4 [61–62] using nonparametric bootstrap (BS) analysis and searches for the best-scoring ML tree in a single run [63]. We performed 1000 rapid bootstrap inferences and, thereafter, a thorough ML search under the GTRCAT model across codon positions.

Additionally, Bayesian inference (BI) analysis was performed using BEAST 1.8.4 [64]. Phylogenetic analyses were conducted in BEAST using the nucleotide substitution models unlinked across codon position and a Birth-Death model with incomplete sampling as tree prior [65]. To determine the model of rate variation among tree branches we first compared the performance of the strict clock and the uncorrelated lognormal clock model using Bayes Factor (BF) in BEAST. Model comparison was performed through a marginal likelihood estimation (MLE) using path sampling (PS) and stepping-stone sampling (SS) with 100 steps of one million iterations each. The uncorrelated lognormal clock model best explained our data (BF_ps_ = 267, BF_ss_ = 273) and was used in the final calibrated analyses.

**Fig 1:**
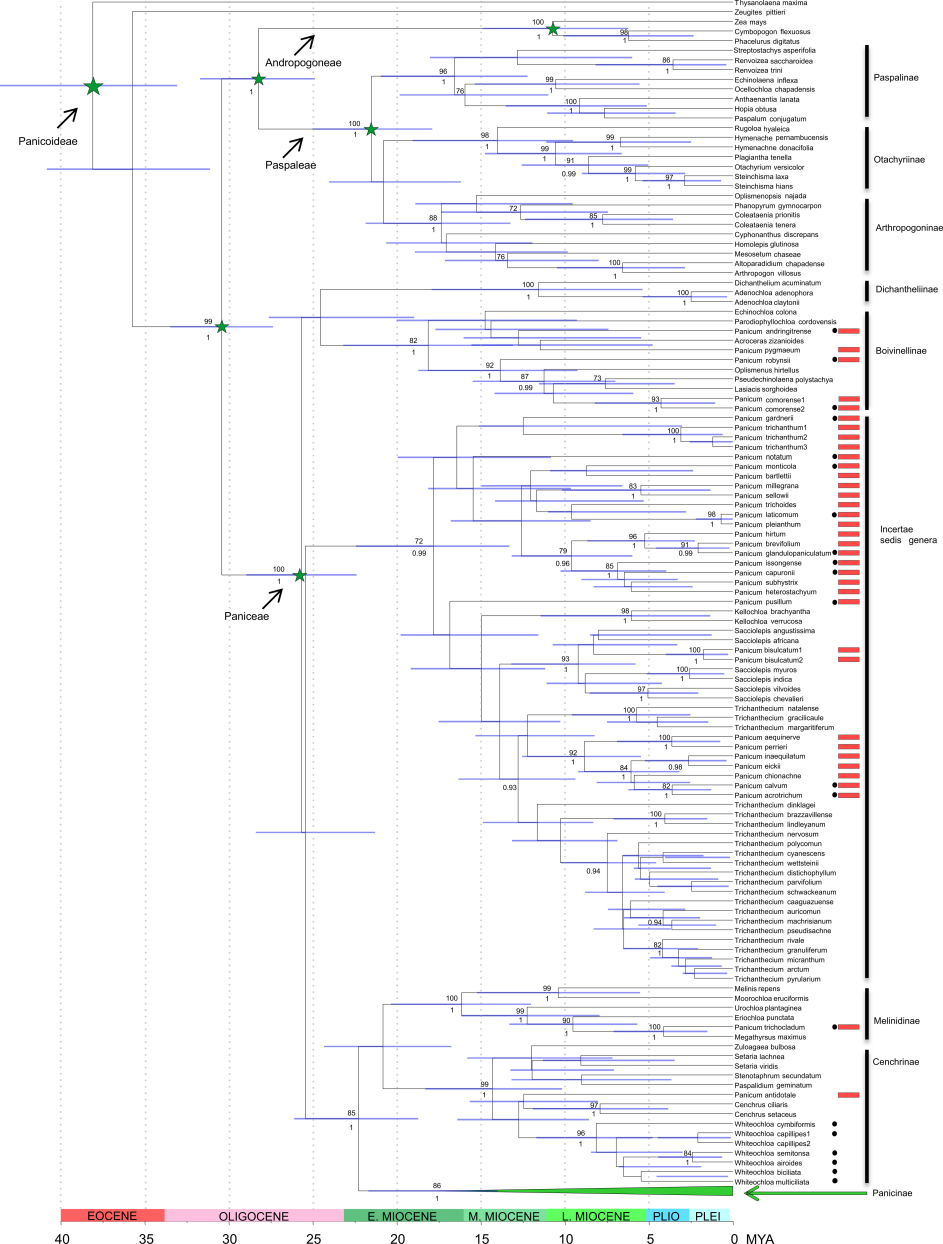
Maximum clade credibility (MCC) tree of Panicoideae obtained from BEAST analyses with the ndhF sequences, using the uncorrelated lognormal relaxed clock model and secondary calibrations based only on external angiosperm fossils (calibration scheme 1). Red boxes indicate phylogenetic placement of Panicum species recovered outside subtribe Panicinae. Maximum likelihood bootstrap ≥ 70% and Bayesian posterior probability ≥ 0.9 are shown above/below the branches, respectively. Horizontal bars on the nodes indicate the 95% HPD of ages. Black circles to the right of taxon names indicate new sequences generated for this study. Subtribe Panicinae are shown in detail in Fig 2. Mya, million years ago; Pli, Pliocene; Plei, Pleistocene. Results from divergence time estimation using the calibration based in the external angiosperm fossils plus grass phytoliths (scheme 2) are shown in Supporting Information S1 Fig.

To estimate divergence times, we used two alternative calibration schemes based on the results of [66]. Because Poaceae has a limited fossil record, and the use of phytolits microfossils [67] strongly affected estimated ages, yielding significantly older estimates, [66] tested two alternative calibration schemes: (1) a calibration based only on external angiosperm fossils (eudicots and non-grass monocots), and (2) a calibration including these fossils together with the controversial phytolith microfossils of Poaceae. The authors concluded that the inclusion of phytolith fossils strongly affect estimated ages and they should be considered only as an alternative to the external calibration, at least until more evidence about their placement becomes available. Based on these results, we used median ages and the 95% high posterior density (HPD) reported by [66] under the two calibration schemes as secondary calibrations in normal prior distributions for the following six crown nodes: subfamily Panicoideae (scheme 1: mean = 38.18 Mya, SD = 3.86) (scheme 2: mean = 48.09.18 Mya, SD = 4.94), most recent common ancestor (MRCA) of supertribes Andropogonodae–Panicodae (mean = 30.31 Mya, SD = 3.27) (mean = 36.65 Mya, SD = 3.64), supertribe Andropogonodae (mean = 28.5 Mya, SD = 3.33) (mean = 34.29 Mya, SD = 3.73), tribe Andropogoneae (mean = 11.79 Mya, SD = 2.95) (mean = 14.45 Mya, SD = 2.71), tribe Paspaleae (mean = 22.6 Mya, SD = 3.13) (mean = 26.25 Mya, SD = 3.61), and supertribe Panicodae (mean = 25.46 Mya, SD = 7.76) (mean = 30.74 Mya, SD = 2.97) (Supertribes and Tribes following classification by [6] Fig 1). BEAUti 1.8.4 was used to generate input files for the analyses, in which substitution models were edited manually on the xml file to fit the models selected using PartitionFinder. We conducted three independent runs of 100 million generations, sampling every 50,000. The first 25% of each run was discarded as burn-in after checking for convergence and effective sample size (ESS) > 200 in Tracer v1.6 [68]. Trees of different runs were then combined using LogCombiner 1.8.4 (http://beast.bio.ed.ac.uk/logcombiner) and the maximum clade-credibility tree (MCC tree) was calculated using TreeAnnotator 1.8.4 (http://beast.bio.ed.ac.uk/treeannotator). Phylogenetic trees were visualized in Figtree v1.4.2. The XML files for BEAST analyses and the trees obtained are available from the Dryad Digital Repository: doi:10.5061/dryad.286gn. All RAxML and BEAST analyses were conducted in the CIPRES Science Gateway v3.3 (http://www.phylo.org/) [69].

### Biogeographic analyses

For biogeographical analyses of Panicinae, we identified seven major areas, modified after [70] and important for the subtribe: (1) North America; (2) South America, including Central America and the West Indies; (3) Eurasia, including Europe, Mediterranean Africa, and temperate Asia; (4) Sub-Saharan Africa, including Madagascar; (5) Southeast Asia, including India, Indo-China, the Malaysian Peninsula, the Philippines, Sumatra, Borneo and the Inner Banda Arc; and (6) Australia, including New Guinea, New Caledonia and New Zealand. Species occurrence data were compiled mainly from extensive examination (conducted by F.O. Zuloaga) of herbarium specimens deposited at B, BA, BAA, BAB, BAF, BR, BRI, C, COL, CORD, CTES, F, G, GH, K, LE, LIL, MA, MEXU, MO, NY, P, SI, US, VEN, W, WIS, herbarium abbreviations from [71], and from the literature, mainly taxonomic revisions, floras, and online databases (GBIF, TROPICOS). Analyses were conducted using the package BioGeoBEARS 0.2.1 [72] implemented in R 3.3.1 [73], which allows comparison of different models of ancestral-area reconstruction. Each model allows for a subset of different biogeographical possibilities, such as dispersal, vicariance and extinction. These biogeographical processes are implemented in an ML framework as free parameters that are estimated from the data [74–75]. We used six different models: DEC, DEC+J, DIVA, DIVA+J, BayArea, BayArea+J (J models include a j parameter controlling founder-event speciation), the maximum number of areas was restricted to the maximum number of regions observed among extant taxa (three) and dispersal probabilities among areas were weighted using a dispersal probability matrix (S1 Table, supplementary material). We did not include temporal stratification in the analyses because divergence of Panicinae was dated from the Miocene and there are not substantial changes in the continental configuration at this time for selected areas [70]. Reconstructions were calculated on the MCC tree inferred in BEAST, pruned to include only the subtribe Panicinae and one specimen per species (except for *P*. *fluviicola* Steud. and *P*. *phragmitoides* Stapf, since they presented alternative phylogenetic placements with PP ≥ 90%). Fit of the models was compared using the Akaike information criterion corrected for sample size (AICc). In addition, and in order to estimate the number and type of biogeographical events (e.g. within-area speciation, vicariance, and dispersal), we used biogeographic stochastic mapping (BSM) [76] under the best fit model (BayArea+j, see results). Event frequencies were estimated by taking the mean and the standard deviation of event counts from 1000 BSMs.

### Evolution of life history

We examined the evolutionary patterns associated with life history in subtribe Panicinae coding the life forms as annual (ie. semelparous) or perennial (ie. iteroparous). Data were obtained from the examination of herbarium specimens, the taxonomic literature, and online databases cited above. Transition rate estimation and ancestral character reconstruction were performed in BayesTraits 2.0.2 [77]. Analyses were conducted employing a continuous-time Markov model of trait evolution with two instantaneous rates representing all possible state changes (q_annual→perennial_ and q_perennial→annual_). Ancestral state reconstructions were executed using the reversible-jump Markov chain Monte Carlo (rjMCMC) method, allowing the analyses to move among different classes of models (for binary traits, five possible models). A reversible-jump hyper prior was set with an exponential prior between 0 and 100, and two independent analyses were run for ten million generation and sampled every 5000 iterations, using 1000 trees randomly subsampled from the posterior distribution of chronograms obtained in BEAST analyses, and pruned to include only the subtribe Panicinae. The first million generations were discarded as burn-in and ESS > 200, while the remaining samples were checked using the R package CODA 0.19–1 [78]. Ancestral states were reconstructed for the MRCA of main sections and clades within Panicinae using the AddMRCA command. Additionally, we compared two models: one in which the rates q01 and q10 were free to vary and another in which rates were constrained to be equal. Fit of the models was evaluated using BF calculated using SS with 100 samples and 10000 iterations per sample.

Numbers of transitions in the life form within Panicinae were estimated using stochastic character mapping (SCM) [79] in phytools 0.6–20 [80] on the 1000 subsampled posterior trees under the best-fitting model (q_01_ = q_10_, ‘ER’ model, see results), 100000 simulations (100 SCM on each of the 1000 trees), and sampling the values of the transition matrix (Q) from its posterior distribution.

Additionally, phylogenetic signal in life history was studied using the method proposed by [81] for discrete (binary) characters, and implemented in the R package caper 0.5–2 [82]. The D-value is estimated as the sum of state changes along branches for a binary trait, with smaller values indicating fewer state changes and supporting the hypothesis that a trait is phylogenetically conserved. We compared the estimated D-value to alternative D values generated with simulated data based on the Brownian evolution threshold model (presence of phylogenetic signal) and the white noise model (no phylogenetic signal). The estimated D-value was then scaled according to the simulated values, such that a D-statistic of 0 indicates the trait conservatism expected under Brownian motion and a value of 1 indicates a random distribution. P values are calculated to determine if the D-statistic is significantly different from simulated D values under the Brownian motion and WN models. We estimated D-values for the 1000 subsampled posterior trees and assessed its significance through 1000 permutations.

## Results

### Phylogeny and divergence times of Panicinae

**Fig 2:**
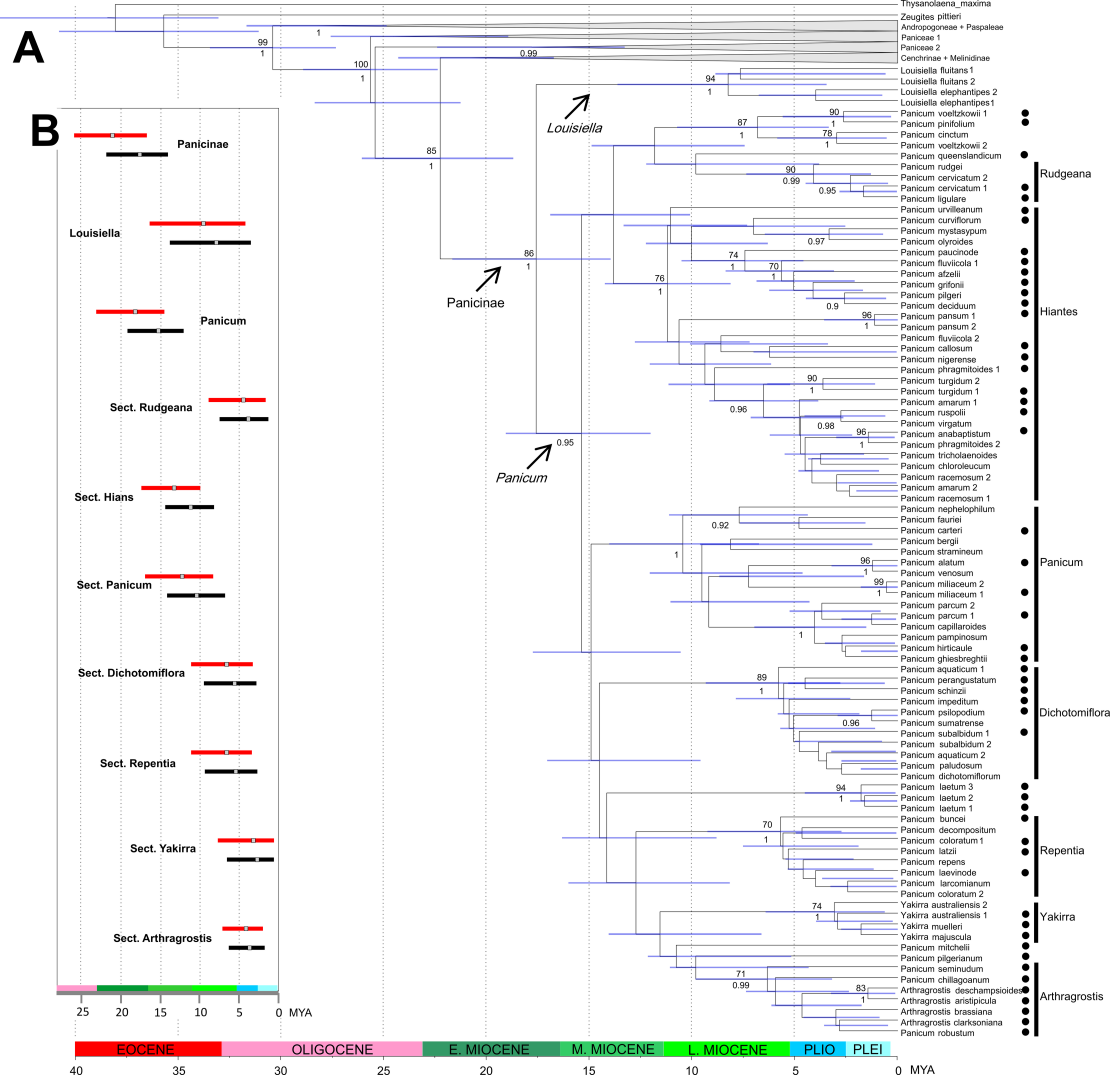
Divergence time estimations for subtribe Panicinae. **A. Maximum clade credibility (MCC) tree of Panicoideae obtained from BEAST analyses with ndhF sequences using the uncorrelated lognormal relaxed clock model and secondary calibrations based only on external angiosperm fossils.** Only subtribe Paniceae is shown in detail; for the remaining clades see Fig 1. Maximum likelihood bootstrap ≥ 70% and Bayesian posterior probability ≥ 0.9 are shown above/below the branches, respectively. Horizontal bars on the nodes indicate the 95% HPD of ages. Vertical bars indicate sections within *Panicum*. Paniceae 1 and Paniceae 2 refer the “Dichanthelinae+ Boivinellinae” clade and the “Incertae sedis genera” clade, respectively. B. Divergence time estimations for crown nodes (MRCA) of subtribe Panicinae, *Louisiella*, *Panicum*, and sections of *Panicum*, based only on external angiosperm fossils (black bars), or angiosperm fossils plus grass phytoliths (red bars). Bars show the 95% HPD of estimated ages, while the squares on bars indicate the median value. Black circles to the right of taxon names indicate new sequences generated for this study. Mya, million years ago; Pli, Pliocene; Plei, Pleistocene.

The analyzed *ndhF* matrix consisted of 214 taxa and 2084 characters, 440 (21%) of which were parsimony informative. The phylogenetic trees recovered from ML and BI analyses were highly congruent (Figs 1–2 and S1–S2 Figs, supplementary material) and recovered subtribe Panicinae [86% bootstrap support (BS), 1.00 posterior probability (PP)] including two main clades: one composed of *Louisiella* [*L*. *elephantipes* (Nees ex Trin.) Zuloaga and *L*. *fluitans* C.E. Hubb. & J. Léonard] and the other including *Panicum* s. str., *Yakirra*, and *Arthragrostis* (Fig 2). The genus *Whiteochloa* was recovered outside the subtribe *Panicinae*, in a strongly supported clade (96% BS, 1.00 PP) within subtribe Cenchrinae. Moreover, 32 species previously assigned to *Panicum* were placed outside Panicinae (Fig 1): four of them (*P*. *pygmaeum* R. Br., *P*. *comorense* Mez, *P*. *andringitrense* A. Camus, and *P*. *robynsii* A. Camus) in subtribe Boivinellinae; *P*. *trichocladum* Hack. ex K. Schum., sister to *Megathyrsus maximus* (Jacq.) B.K. Simon & S.W.L. Jacobs in subtribe Melinidinae; *P*. *antidotale* Retz. remains in Cenchrinae; and the remaining 26 species appear distributed in the *Sacciolepis*-*Trichanthecium*-*Kellochloa* clade of tribe Paniceae. Finally, within the *Panicum* s. str. clade of Panicinae (Fig 2) seven well supported groups were recovered, representing different sections of *Panicum*: *Rudgeana* (Hitchc.) Zuloaga (BS: 90, PP: 0.99), *Hiantes* Stapf (BS:76, PP: 1.00), *Panicum* (BS: 67, PP: 1.00), *Dichotomiflora* (Hitchc.) Hitchc. & Chase ex Honda (BS: 89, PP: 1.00), *Repentia* Stapf (BS: 70, PP: 1.00), and genera *Arthragrostis* (BS: 71, PP 0.99) and *Yakirra* (BS: 74, PP: 1.00) (Fig 2 and S2 Fig of supplementary material).

Divergence time analyses using the calibration scheme based on the external angiosperm fossils of [66] (scheme 1) recovered younger estimates than analyses including the phytoliths (scheme 2) (Table 2, Fig 2 and S2 Fig of supplementary material). However, results from both schemes dated the crown node of subtribe Panicinae principally in the Early Miocene (scheme 1: 17.55 Mya, 95% HPD 21.68–13.98; scheme 2: 21.04 Mya, 95% HPD 25.82–16.73). Within Panicinae, the MRCA of *Panicum* was estimated around Early-Mid Miocene (15.27Mya, 95% HPD 19.07–12.03; 18.15 Mya, 95% HPD 23.04–14.51). MRCAs of sects. *Hiantes* and *Panicum* were estimated around Mid-Late Miocene, while MRCAs of the remaining sections diversified principally during the late Miocene to Pliocene. Table 2 and Fig 2 and S2 Fig provide node ages (median and 95% HPD) for main clades in Panicinae. Subsequent studies were conducted with results obtained from the analyses under scheme 1.

**Table 2:** Estimated ages (Mya; median and 95% HPD) for MRCA of the main clades within the subtribe Panicinae using the two alternative calibration schemes (scheme 1: Calibration based only on external angiosperm fossils, scheme 2: Calibration including these fossils together with the phytolith microfossils of Poaceae), and their corresponding support values (PP: Bayesian posterior probability).

	Calibration scheme			
	1		2	
clade (MRCA)	Median (95% HPD)	Support (PP)	Median (95% HPD)	Support (PP)
Subtribe Panicinae	17.55 (21.68–13.98)	1	21.05 (25.82–16.73)	0.99
*Louisiella*	7.93 (13.64–3.46)	1	9.49 (16.3–4.21)	1
*Panicum*	15.27 (19.07–12.03)	0.95	18.15 (23.04–14.51)	0.94
Sect. *Rudgeana*	3.83 (7.37–1.3)	0.99	4.48 (8.72–1.68)	0.99
Sect. *Hiantes*	11.13 (14.26–8.14)	1	13.23 (17.3–9.93)	0.99
Sect. *Panicum*	10.37 (14.04–6.75)	1	12.28 (16.81–8.3)	1
Sect. *Dichotomiflora*	5.6 (9.34–2.79)	1	6.58 (10.93–3.26)	1
Sect. *Repentia*	5.5 (9.26–2.74)	1	6.58 (10.98–3.41)	1
Sect. *Yakirra*	2.75 (6.43–0.63)	1	3.22 (7.63–0.54)	1
Sect. *Arthragrostis*	3.65 (6.15–1.75)	0.99	4.23 (7.01–1.96)	0.99

### Biogeographical analyses

**Fig 3:**
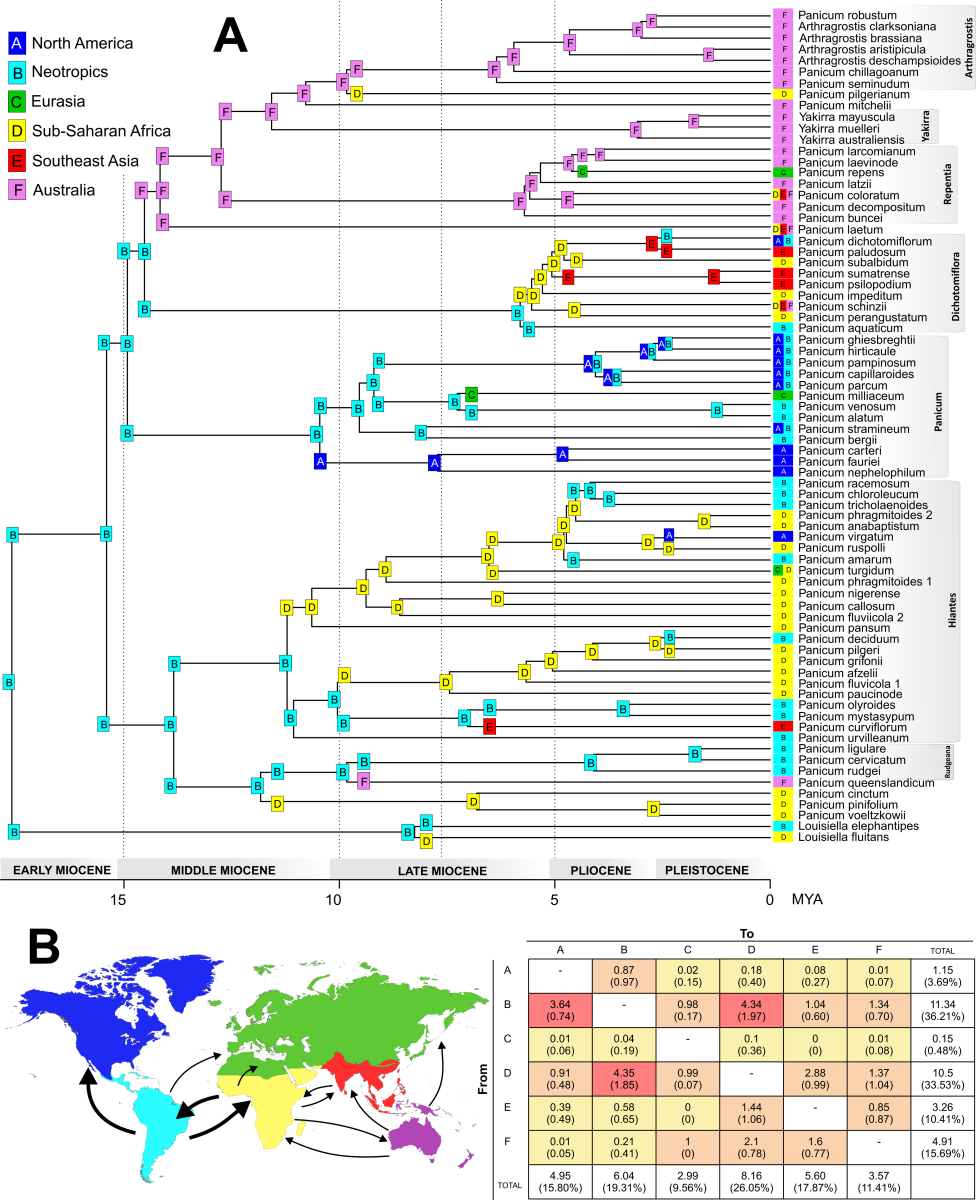
Biogeography of subtribe Panicinae. A. Ancestral range estimation (ARE) on the Panicinae chronogram using the BayArea+J model in BioGeoBEARS. States at nodes (squares) represent the area with highest ML probability before the instantaneous speciation event, whereas those on branches represent the state of the descendant lineage immediately after speciation. Squares with more than one letter refer to ancestral areas composed of more than one biogeographical area. Branch labels have been removed to reduce overlap in cases where they are identical to the state at both the ancestral and the descendant node. Boxes to the left of taxon names indicate areas of tip species. S2 Fig of supplementary material provides all ARE per node and corner with pie charts representing probability of each ancestral area. B. Results from 1000 biogeographic stochastic mapping (BSM) under the BayArea+J model in BioGeoBEARS. Numbers of dispersal events (range-expansion dispersals plus cladogenetic founder/jump dispersal) among areas for Panicinae. Counts of dispersal events were averaged across the 1000 BMSs and are presented here with standard deviations in parentheses. Colour temperature indicates the frequency of events. The sum and corresponding percentages of events involving each area, either as a source for dispersal (the rows) or as a destination (the columns). Map on the left shows main dispersal routes recovered in the BSM analyses. Thick arrows correspond to more frequent dispersal routes.

Of the six biogeographical models evaluated using BioGeoBears, the BayArea+j model resulted the best supported (AICc_wt_ ~ 1, Table 3). The inclusion of the “jump dispersal” parameter j significantly improved all models (BayArea+j, DEC+j, and DIVA+j) (Table 3), suggesting for Panicinae that the models without founder-event speciation (only accounting for dispersal via anagenetic range expansion) are not adequate to account for all movements to new areas. Ancestral range estimation under the BayArea+J model (Fig 3 and S3 Fig of supplementary material) suggests the Neotropics as the most probable ancestral area of the MRCA of the Panicinae (p = 0.78) and its early diversification during the Early-Mid Miocene, including the MRCA of *Panicum* s. str. (p = 0.76). Subsequent diversification of main clades from the Mid-Miocene to Pliocene involves four primary biogeographical routes: 1) Neotropic- Sub-Saharan Africa, 2) Neotropic-North America, 3) Sub-Saharan Africa- Southeast Asia, 4) Australia-Old World. Clades representing the genus *Louisiella*, *Panicum* incertae sedis, and sects. *Rudgeana* and *Hiantes* diversified primarily in the Neotropics and Sub-Saharan Africa, with several dispersal events between these two areas (Fig 3). Diversification in sect. *Panicum* mostly occurred between the Americas (North America and Neotropics). In the remaining clades of *Panicum* s. str. the Americas were poorly-represented. Diversification in sect. *Dichotomiflora* involved Sub-Saharan Africa with dispersals to Southeast Asia, whereas in sect. *Repentia* and genera *Yakirra* and *Arthragrostis* ancestral areas were mainly in Australia, with subsequent dispersal to Sub-Saharan Africa, Southeast Asia, or Eurasia.

**Table 3:** Comparison of the fit of the models tested in BioGeoBEARS, all including or not founder-event speciation (“+j”). Log-likelihood ln(L), Akaike information criterion corrected for sample size (AICc), difference in AICc value compared with the best model (ΔAICc), and the Akaike weights (ωi) showing the relative likelihood of each model.

Model	LnL	AICc	ΔAICc	ω_i_
DEC	-153.25	310.67	49.43	0.00
DEC+j	-137.21	280.76	19.12	0.00
DIVA	-153.96	312.10	50.86	0.00
DIVA+j	-137.57	281.47	20.23	0.00
BayArea	-159.47	323.11	61.87	0.00
BayArea+j	-127.45	261.24	0	1.00

BSM analyses revealed that biogeographical events in the Panicinae comprise within area-speciation (63% of total events) and dispersals (37%), of which 12% correspond to range-expansion dispersals (anagenetic dispersal) and 25% to cladogenetic dispersals (cladogenetic founder/jump dispersal) (S2 Table, supplementary material). Within area-speciation was greater in Africa and Australia and lower in Southeast Asia and North America. Regarding dispersal events, the highest number of dispersals involved interchanges between the Neotropics and Sub-Saharan Africa (Fig 3B), mainly within *Louisiella*, sect. *Rudgeana*, and sect. *Hiantes*, followed by movements, mostly in sect. *Panicum*, from the Neotropics to North America. Overall, the Neotropics were the most common source for the estimated dispersal events (ca. eleven of 31 events, 36%), whereas Sub-Saharan Africa resulted the largest destination (approx. eight events, ~26%) (Fig 3B).

### Evolution of life history

**Fig 4:**
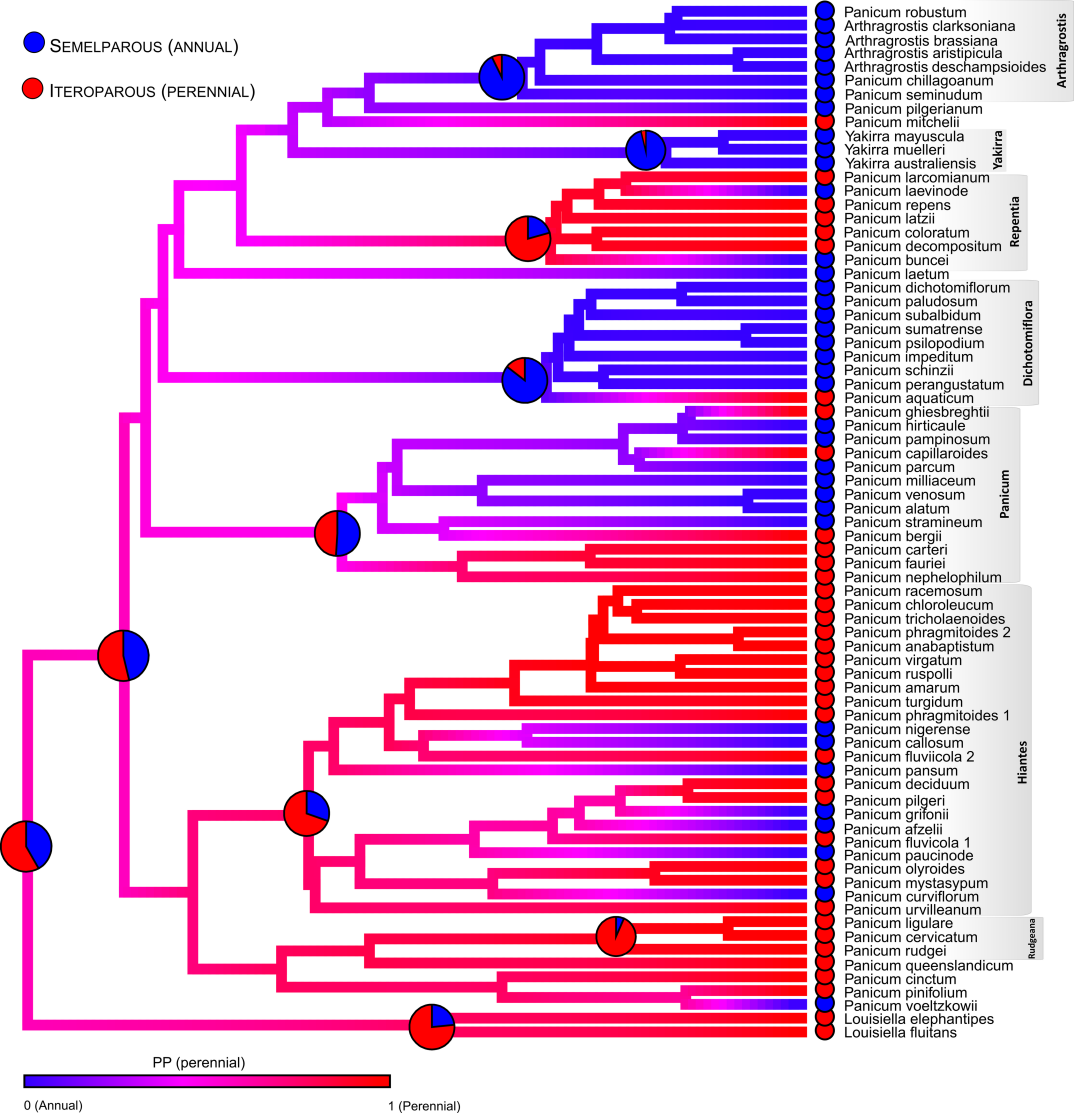
Result from 100 000 stochastic character-mapping reconstructions of life history (annual vs. perennial) on the MCC tree of the subtribe Panicineae using Phytools. The colour of edges in the tree gives the posterior probability (computed as the relative frequency across stochastic maps) of each habit type through the branch of the clade. Red indicates high posterior probability of perennial habit. Pie charts on the main nodes of the Panicineae show character-state probability (blue, annual; red, perennial) from reconstructions in BayesTraits using the 1000 subsampled posterior trees and the rjMCMC method.

Analyses of habit evolution in Panicinae using the rjMCMC showed that the model with the highest marginal probability was an Equal Rates model (p = 0.97), with the probability of change from perennial to annual the same as the probability of reversal. This model (q_01_ = q_10_) was also strongly supported by the BF over the two-rates model (BF_q01 = q10/ q01,q10_ = 8.24). Ancestral state reconstruction (Fig 4) favored the annual habit for the MRCA of genera *Yakirra* (p = 0.96), *Arthragrostis* (p = 0.93) and section *Dichotomiflora* (p = 0.85); and perennial habit in MRCA of *Louisiella* (p = 0.77), and sects. *Rudgeana* (p = 0.93), *Hiantes* (p = 0.70) and *Repentia* (p = 0.79). Reconstructions for the MRCA of subtribe Panicinae, *Panicum* s. str., and section *Panicum* were ambiguous. The transition count between the two states over the 100,000 SCMs for Panicinae recovered a median of 42 total changes in the life history, with 21 from annual to perennial and 22 for the reverse shift. SCM analyses also indicate that the mean total evolutionary time of Panicinae associated with the annual and perennial habit was similar (47% and 53%, respectively).

Phylogenetic signal estimation for the life form over the 1000 subsampled posterior trees using the Fritz and Purvis’ D statistic resulted in a mean value of 0.18 (95% quantiles 0–0.36), recovering a significant phylogenetic signal (100% of the trees rejected the white noise model with p<0.01), and not significant differences from the distribution expected under a Brownian threshold model (only 0.3% of trees rejected the BM model with p<0.05).

## Discussion

Although our analysis was only based on the *ndhF chloroplast gene*, the results are completely congruent with previous multilocus plastid phylogenies of this group [15, 52, 55]. Due to the potential pitfalls related to single-locus analyses (see materials & methods), new multilocus phylogenies, both with nuclear and plastid data, are needed to confirm results obtained here. Nevertheless our findings represent a major step in understanding the systematics and evolution of *Panicum*.

### Implications for systematic and taxonomy

Results of our study stress that subtribe Panicinae includes only two genera, *Louisiella* and *Panicum*, in agreement with [6]. Species of *Whiteochloa* remaining in subtribe Cenchrinae, together with “*P*.*” antidotale* Retz.; one species, “*P*.*” trichocladum*, in the Melinidinae; and non-Kranz “*Panicum*” species in different positions of subtribes Boivinellinae, and in the *incertae sedis* clade of the Paniceae. From now on “P” or “*Panicum*” species designates what we consider non *Panicum* species.

#### Kranz genera and species excluded from *Panicum*

*Whiteochloa*. This genus includes six species and was classified within subtribe Cenchrinae [3, 5–6, 29], with the analyzed species sister, with low branch support, to the genera *Pseudoraphis* Griff and *Chamaeraphis* R. Br. [53], in a study of Panicoideae based on plastome phylogenomics, discussed the position, with maximum support, of *W*. *capillipes* within the Panicinae. Nevertheless, these authors were cautious and did not reclassify the genus since they analyzed only one species of the genus. During the current analysis, we studied all species of *Whiteochloa*, which appeared in a strongly supported clade within subtribe Cenchrinae, confirming previous studies and its position in this subtribe [3, 5–6, 29].

“*Panicum” trichocladum* Hack. ex K. Schum. This species belongs to subtribe Melinidinae, where it is related, in a strongly supported clade, to *Urochloa*, *Eriochloa*, and *Megathyrsus*. Most species of *Eriochloa* and the genus *Megathyrsus* were included as synonyms of *Urochloa* by [16], while [6] considered valid, as traditionally circumscribed, the three genera. Therefore, and taking into account that the limits of the included genera in Melinidineae are far from clear [16], we do not attempt to place “*P*." *trichocladum* in a particular genus within the Melinidinae.

#### Non-Kranz species excluded from *Panicum*

Although many species of *Panicum* s.l. were studied in previous treatments (see literature cited in Table 1) we have added more than ten additional taxa. As a result, *“P*.*” pygmaeum*, *“P*.*” robynsii*, *“P*.*” comorense* and *“P*.*” andringitrense* belong to subtribe Boivinellinae (mostly C_3_), consistent with new results by [15], who also considered within this clade other species, such as *“P*.*” ibitense* A. Camus, *“P*.*” cupressifolium* A. Camus, *“P*.*” andringitrense*, *“P*.*” vohitrense* A. Camus, and “*P*.*” malacotrichum* Steud. Relationships of these taxa within this subtribe are still unclear and need further study. Another group of non Kranz species (classified in Group 1 [20], author who analyzed the anatomy of “*P*.*” laticomum* Nees, *“P*.*” monticola* Hook. f., *“P*.*” heterostachyum* Hack., *“P*.*” aequinerve* Nees, and “*P*.*” brevifolium* L.) are, with strong support but also in need of a complete analysis, in the *incertae sedis* clade of the Paniceae composed of *Sacciolepis* Nash, *Trichanthecium* Zuloaga & Morrone, *Kellochloa* Lizarazu, M.V. Nicola & Scataglini, and “*Panicum”* sect. *“Monticolae”* [16], all C_3_. Within this clade, *“P*.*" notatum* Retz., *“P"*. *gardneri* Thwaites, *“P*.*" monticola*, *“P*.*" pleianthum* Peter, and *“P*.*" laticomum* are in an unresolved position, while *“P*.*” glandulopaniculatum* Renvoize, *“P*.*” subhystrix* A. Camus, *“P*.*” issongense* Pilg., and *“P*.*” capuronii* A. Camus appeared in a strongly supported clade with no clear morphological synapomorphies [16], together with “*P*.*” brevifolium*, *“P*.*” hirtum* Lam., and “*P*.*” heterostachyum*. Finally, several species are also in a strongly supported clade embedded in the genus *Trichanthecium*; they are *“P*.*” perrieri* A. Camus, *“P*.*” acrotrichum* Hook. f., *P*. *calvum* Stapf, *“P*.*” aequinerve*, *“P*.*” chionachne* Mez, *“P*.*” inaequilatum* Stapf & C.E. Hubb., *“P*.*” eickii* Mez (the latter four species mentioned in this position by [17]), while *“P*.*” pusillum* Hook. f. is a sister species of *Kellochloa*. A similar result was presented [15] for *“P*.*” perrieri* A. Camus and “*P*.*” ambositrense* A. Camus. The inclusion of these species in *Trichanthecium* will require a recircumscription of the genus, a goal beyond the scope of this contribution.

#### Subtribe Panicinae and *Panicum* s. str

Subtribe Panicinae forms a strongly supported clade; morphologically, its taxa are defined mainly by having an open to lax panicles, spikelets dorsiventrally compressed, upper anthecium indurate and convex, a basic chromosome number of *x* = 9, with all species Kranz of the NAD-me subtype. This subtribe comprises the genera *Louisiella* and *Panicum*, the latter also including as synonyms *Arthragrostis* and *Yakirra*.

***Louisiella*.** Both species of *Louisiella* are aquatic perennials with spongy culms, leaves lanceolate, flat, inflorescence lax and open, with spikelets lanceolate, the lower glume 1/6 its length, nerveless to 1-nerved, lower flower absent, and caryopsis with a linear hilum. Both species have the outer parenchymatous bundle sheaths with centrifugally arranged specialized chloroplasts, a feature also distinguishing species of *Panicum* sect. *Dichotomiflora* [20]; they also include four molecular synapomorphies at positions 279, 540, 1,260 and 1,431 of the *ndhF* matrix [50].

***Panicum* sect. *Repentia*** This clade includes *Panicum repens* L. and another six species, mostly cespitose perennials with stout rootstocks, spikelets clustered toward the branch tips, with the lower glume ¼ to 1/3(-1/2) the length of the spikelet, and lower palea and lower flower usually present. Species of sect. *Repentia* were treated within group Virgata [83–84], in sect. Repentes Stapf (including *P*. *repens*), and sect. Coloratae Stapf [85] with *P*. *coloratum* L. [86] considered in sect. *Repentia* species of this clade, i.e., *P*. *repens*, together with species of clade Hiantes; later, [18] placed sect. *Virgata* Nees in synonymy of sect. *Repentia* (see comments under clade Hiantes), although he mentioned that species of *Virgata* and *Repentia* could be classified in two different groups. Recently, [26] classified *P*. *repens* and allied species within sect. *Dichotomiflora*.

***Panicum* sect. *Yakirra*** This is the strongly supported *Yakirra* lineage, congruent with several molecular studies that have found *Yakirra* to be nested within *Panicum* [3, 16, 50, 57]. This genus was segregated from *Panicum* by [87], based on *Panicum pauciflorum* R.Br. [*Yakirra pauciflora* (R. Br.) Lazarides & R.D. Webster], in order to include a group of Australian species that were previously transferred from *Panicum* to *Ichnanthus* [88]. *Yakirra* was distinguished by the presence of a stipitate upper anthecium, as well as a prominent rachilla between the upper glume and lower lemma. The stipe at the base of the upper anthecium in *Yakirra* has a pair of winged appendages [57], described by [89] as a swollen stipe with two acute lobes. As [57] pointed out, a stipe and elongate rachilla are also present in species of *Panicum* s.str., such as those of *Panicum* sect. *Rudgeana*, and also, as previously mentioned, in species now considered within sect. *Arthragrostis*. Our analyses confirm the inclusion [16] of *Yakirra* within *Panicum* s.str.

***Panicum* sect. *Arthragrostis*.** We find strong support for a lineage corresponding to species previously treated in the genus *Arthragrostis*. This genus was established by [90], based on *Panicum deschampsioides* Domin [*Arthragrostis deschampsioides* (Domin) Lazarides]. This author characterized the genus by the disarticulation of the inflorescence into component divisions, the stipitate upper anthecium, and the presence of a conspicuous rachilla between the lower and upper glume. Our study grouped seven species, four of them previously treated as *Arthragrostis*, *A*. *aristispicula* (= *Panicum aristispiculum*), *A*. *brassiana* (= *P*. *brassianum*), *A*. *clarksoniana* (= *P*. *clarksonianum*), and *A*. *deschampsioides* in a strongly supported clade together with *P*. *seminudum*, *P*. *chillagoanum* and *P*. *robustum*, while *P*. *mitchelii*, and *P*. *pilgerianum* appeared as the sister taxa of this clade; *P*. *mitchelii* is a perennial species with the rachilla internodes elongated between the glumes but no stipe present at the base of the upper anthecium, while *P*. *pilgerianum* is an African annual and aquatic species, with lanceolate spikelets, the lower glume 1/6 to 1/5 the length of the spikelet, without a morphological relationship with species of *Arthragrostis*. The morphological characters that defined *Arthragrostis* also appear in some species of *Panicum*. The inflorescence disarticulates at maturity in as *P*. *bergii* Arechav. and *P*. *olyroides* Kunth; similarly, several species, i.e., *P*. *ligulare* Nees ex Trin., *P*. *cervicatum* Chase, among others, have a conspicuous internode between the glumes and below the upper anthecium. Therefore, we are considering *Arthragrostis* as a section within *Panicum*, including seven Australasian species, characterized as being annuals with open and lax inflorescences, disarticulating or not at maturity, with spikelets ovoid to lanceolate, glabrous, occasionally pilose, the rachilla conspicuous between the lower and upper glume, and upper anthecium stipitate or not, indurate.

***Panicum* sect. *Dichotomiflora*.** We refer to this lineage as the *P*. *dichotomiflorum* Michx. complex, since this species is the most wide-ranging member of this group in the New and Old Worlds. All species in this clade share the following morphological characteristics: annuals, occasionally perennial, with soft culms, blades flat, lanceolate; inflorescence with the branchlets short and appressed, spikelets glabrous, the lower glume 1/5 to ¼ the length of the spikelet, the upper glume and lower lemma subequal, (5-)7-9(-11) nerved, growing in wet and open places. [20] classified species, here arranged in sect. *Dichotomiflora*, i.e., *P*. *schinzii* Hack., *P*. *subalbidum* Kunth, in a group of species with PEP-ck type anatomy, i.e., with a Kranz outer bundle sheath in which the specialized chloroplasts are centrifugally arranged. Taxa here considered were treated in group Dichotomiflora [83–84], including *P*. *elephantipes* Nees ex Trin. (= *Louisiella elephantipes*), and subsequently in sect. *Dichotomiflora* [18, 91]. On the other hand, [26] grouped in sect. *Dichotomiflora* some species, i.e., *P*. *repens* and *P*. *coloratum*, that are placed here in sect. *Repentia* and differ from sect. *Dichotomiflora* by having conspicuous rootstocks and distichous, involute leaves. [20] also pointed out that *P*. *repens* differs anatomically from species here classified in sect. *Dichotomiflora*, by cross-sectional anatomy and epidermal structure. Species in this clade are widely distributed in southeast Asia, Africa, and North and South America.

***Panicum* sect. *Rudgeana*.** This is a strongly supported clade which includes three species of sect. *Rudgeana* [92], *P*. *ligulare* Nees ex Trin., *P*. *cervicatum* Chase, and *P*. *rudgei* Roem. & Schult. Morphologically, they are cespitose plants with erect culms, membranous-ciliate ligules, blades lanceolate to linear-lanceolate, inflorescence a terminal, open and lax panicles, spikelets pilose or glabrous, with the lower glume ½ to ¾ the length of the spikelet, and upper anthecium stipitate, the stipe membranous ventrally and indurate dorsally. All five species of this section grow in open places in Central and South America and differ morphologically from species of sects. *Arthragrostis* and *Yakirra*: species of sect. *Arthragrostis* have a conspicuous rachilla between the lower and upper glume and a homogeneous and slender stipe is present below the upper anthecium. On the other hand, species of sect. *Yakirra* present a swollen stipe with two acute lobes below the upper anthecium [57, 87].

As sister species of this clade we found *P*. *queenslandicum* Domin, an Australian perennial species with a small stipe at the base of the upper anthecium.

***Panicum* sect. *Panicum*.** We find strong support for a lineage corresponding to the *P*. *miliaceum* complex, treated here as *P*. sect. *Panicum* as recognized by previous authors [3, 16, 18, 26, 29]. Within this clade, three species from Hawaii, *P*. *carteri* Hosaka, *P*. *fauriei* Hitchc., and *P*. *nephelophilum* Gaudich. grouped together in a supported clade. Morphologically, species of sect. *Panicum* are characterized by being annual or cespitose perennials, with culms erect, inflorescences open and lax, bearing terminal spikelets with the lower glume (1/3-)1/2-3/4(-4/5) the length of the spikelet, 3-5(-9) nerved, and upper glume and lower lemma (5-)7-9(-15) nerved, the spikelets without elongated or modified rachilla internodes. [20] described *P*. *miliaceum*, and other “true” *Panicum* species, as having double bundle sheaths and centripetally located Kranz chloroplasts. This lineage is widely distributed in the New World, Africa, Asia, Australia, and the Pacific.

***Panicum* sect. Hiantes.** This clade includes a group of American and African and Asian species with strong support; morphologically, they are characterized as annual or perennial, cespitose species, with a terminal, lax and open to contracted panicles, spikelets gaping at maturity, with the lower glume ¾ to 4/5 the length of the spikelet, and lower palea and lower flower present. Species of this clade traditionally were classified in groups Urvilleana and Virgata [83–84], together with species of Repentia), in sects. *Hiantes* and *Dura* [85] in Africa, in sects. *Repentia* and *Urvilleana* [18], with *P*. *olyroides* Kunth as an ungrouped species of *Panicum*) in the New World, and in sects. *Urvilleana* and *Virgata* [26]. Recently [53] also considered sect. *Urvilleana* within sect. *Virgata* (= *Hiantes*). *Panicum chloroleucum* Griseb., *P*. *racemosum* (P. Beauv.) Spreng., and *P*. *urvilleanum* Kunth, American species previously grouped in sect. *Urvilleana*, are all perennial species with conspicuous rhizomes, and spikelets pilose, with the upper lemma with long macrohairs toward its base. *Panicum olyroides* Kunth and *P*. *mystasipum* Zuloaga & Morrone, previously ungrouped species within *Panicum*, are also, in a strongly supported clade, within sect. *Hiantes*, with *P*. *curviflorum* Hornem., an Asian species, as sister taxon of this clade.

***Panicum* incertae sedis clade.** This clade consists of three species, two of them, *P*. *voeltzkovii* A. Camus and *P*. *cinctum* Hack., endemic to Madagascar; both are erect cespitose perennials, with linear to lanceolate, flat, blades, inflorescence open and terminal, and spikelets ovoid, with the lower glume ½ its length and the upper glume and lower lemma subequal, 5-7-nerved. Our result agrees with that of [15], who showed both species, together with *P*. *luridum* Hack. in a strongly supported clade. Our analysis differs by the presence, in this clade, of *P*. *pinifolium* Chiov., a species morphologically similar to *P*. *repens* with linear to aciculate, distichous leaves, and lower glume reduced, nerveless to 1-nerved.

***Panicum* incertae sedis species.** *Panicum laetum* appears ungrouped and sister to the clade including sects. *Repentia*, *Arthragrostis* and *Yakirra*. This is an annual species growing in Africa and Asia characterized by its open and lax panicles, spikelets with the lower glume ½ or more the spikelet length, lower flower absent, and upper glume and lower lemma 7-9-nerved.

### Spatio-temporal diversification of *Panicum*

Results obtained here suggest that early diversification of *Panicum* occurred through the Early-Mid Miocene in the Neotropics, and principally during the warm period of the Mid-Miocene climatic optimum [93]. Divergence time analyses [66] did not included members of subtribe Panicinae; nevertheless our age estimations for other panicoid groups were in general consistent with those reported by them and other authors [27–28, 54, 94]. [15] recovered the crown node of *Panicum* s. str. around the Mid-Miocene (~ 12 Mya), while estimates of [27–28] placed it in the Late Miocene (6–8 Mya), after the mid-Miocene climatic optimum, when the global climate became cooler. However, *Panicum* s. str. and subtribe Paniceae in these later phylogenies are poorly represented (below 5%).

Dispersal events seem to have played an important role in the biogeographic diversification of *Panicum*. The importance of dispersal in panicoid and other grasses was reported in the extensive biogeographical analyses on grass diversification in Madagascar presented by [15]. These authors concluded that the extant grass flora in Madagascar was the result of multiple overseas dispersals. In *Panicum* s. str., dispersals were recovered as the most frequent biogeographic event for range change, both involving anagenetic dispersal (i.e., range expansion) and cladogenetic dispersal (i.e., founder-event speciation) [75]. Diversification of sections *Hiantes* and *Panicum*, for which a Mid-Miocene origin was estimated, were characterized by two main dispersal routes from the Neotropics, to Sub-Saharan Africa for the former, and North America for the latter. The second group of sections/genera recovered within *Panicum*, including sects. *Rudgeana*, *Repentia*, *Dichotomiflora*, and genera *Arthragrostis* and *Yakirra*, exhibited younger divergences, with their crown node ages recovered mainly through the Late Miocene–Pliocene, their diversification associated with gradual global cooling. In these groups, interchanges with America are infrequent, with the exception sect. *Rudgeana*. Section *Dichotomiflora* seems to have dispersed from the Neotropics to Africa by the end of the Late Miocene (around 5.6 mya), exhibiting subsequent dispersions principally to Southeast Asia in the Pliocene. For the MRCA of sect. *Repentia*, and genera *Arthragostis* and *Yakirra*, the Australian continent was recovered as the most likely ancestral area, involving the oldest dispersal from the Neotropics in the Panicinae, most likely around the Mid-Miocene (~13 Mya).

### Evolutionary patterns of the life history in *Panicum*

Our analyses show that rates and changes between annual and perennial life history in *Panicum* s. str. were quite frequent and similar, suggesting considerable lability of life history and the absence of strong evolutionary constraints. Evolutionary labile traits related to niches have been associated with different intrinsic factors including genetic variation, biophysical constrains, epistatic interactions, and complexity of the new phenotypes [95–98]. This evolutionary lability of the life history in the subtribe Panicinae and *Panicum* s. str., added to the presence of C_4_ photosyntesis, could have facilitated repeated shifts between habitats and the colonization of new areas. Evidence reported by [99] suggests that changes from C_3_ to C_4_ photosynthesis among panicoid grasses promoted niche expansion into hotter climates, and also into more arid climates for tribe Paniceae. These traits, together with the numerous dispersal events since the Late Miocene, could have generated the widespread distribution of the group.

Transitions between annual and perennial growth habit are reported to be associated mainly with temperature. Annuals are favored in hot conditions with highly variable and unreliable precipitation patterns, and in disturbance regimes, both of which can adversely affect adult perennial plants [100–102]. Perennials are favored in colder environments with short growing seasons [64, 103–104]. Thus, annuals are common in desert floras and are apparently better adapted than perennials to lowland areas with greater temperatures, while perennials are better adapted to cooler environments, principally in alpine habitats [105–106]. However, *Panicum* species, both annuals and perennials, are distributed in tropical and temperate lowlands, and they rarely occur at latitudes or elevations with short growing seasons. Therefore, further empirical analyses using georeferenced specimen data and aridity index values together with potential evaporation (PET), and other variables related to the ecological niche, should be conducted within *Panicum* s. str. to detect the ecological correlates of life history traits in this group.

## Concluding remarks

Our analyses support the circumscription of subtribe Panicinae as comprising two genera: *Louisiella* and *Panicum*, while *Arthragrostis* and *Yakirra* are treated as synonyms of *Panicum*. Also, this study supports the recognition of seven sections in *Panicum*. Nearly 40 non-Kranz species belong in other subtribes of Paniceae, and one Kranz species goes to Cenchrinae and another to Melinidineae. Evidence obtained here suggest that the early diversification of *Panicum* s. str. occurred in the Early to Mid-Miocene, while subsequent diversification of its sections took placed mainly through the Late Miocene–Pliocene. Recurrent dispersals, together with the considerable lability of the life-form, along with the advantages of C_4_ photosynthesis, seem to have favored the widespread distribution and diversification of the genus in latitudes with hot dry, and warm wet, long growing seasons.

## Taxonomic treatment

**Subtribe *Panicinae*** Fr., Fl. Scan.: 195. 1835. Type. *Panicum* L.

Annual or perennial; ligules membranous-ciliate to ciliate. *Blades* oblong-lanceolate to linear-lanceolate. *Inflorescence* an open and lax panicle. *Spikelets* dorsiventrally compressed, the lower glume reduced or up to the full length of the spikelet; upper glume and lower lemma subequal; upper anthecium indurate, abaxially convex, with simple or compound papillae toward the apex. *Caryopsis* with a linear to punctiform hilum. Basic chromosome number *x* = 9. Photosynthetic pathway; C_4_ subtype, NAD-me.

Including two genera, *Louisiella* and *Panicum*, distributed worldwide mainly in tropical and subtropical areas.

**Louisiella** C.E. Hubb. & J. Léonard, Bull. Jard. Bot. État Bruxelles 22: 316. 1952. Type species. *Louisiella fluitans* C.E. Hubb. & J. Léonard

Aquatic perennials, with succulent culms and internodes spongy. *Blades* oblong-lanceolate to linear-lanceolate. *Spikelets* lanceolate, glabrous, lower glume reduced, hyaline, nerveless to 3-nerved, upper glume and lower lemma longer than the upper anthecium, (5-)7-9 nerved, lower palea reduced or absent, lower anthecium flower absent; upper anthecium not stipitate, shiny. *Caryopsis* with a linear hilum.

Genus with two species, present in tropical and subtropical areas of America, *L*. *elephantipes*, and Africa, *L*. *fluitans*.

**Panicum** L., Sp. Pl.: 55. 1753. Type species. *Panicum miliaceum* L.

Annual or perennial, mostly cespitose, with culms erect to decumbent and rooting and branching at the lower nodes. *Blades* lanceolate to linear-lanceolate. *Inflorescence* a terminal open panicle, axillary inflorescences occasionally present. *Spikelets* pedicelled on second or third-order branches, pilose or glabrous, the rhachilla conspicuous or not between the bracts, with the lower glume ¼ to 4/5 the length of the spikelet, 3-9-nerved; upper glume and lower lemma usually subequal, (5-)7-11(-13) nerved; lower anthecium palea and lower flower present or absent; upper anthecium stipitate or not, indurate, often textured, shiny. *Caryopsis* with a punctiform hilum.

A pantropical genus with nearly 163 species worldwide and classified in seven sections (Table 4). Of these sections, one is endemic to Australia, another is present in Australia and southeast Asia, one is restricted to the Neotropics, and the other four are pantropical. Non *Panicum* species are listed on Table 5.

**Table 4:** Preliminary list of species of *Panicum* by section and ungrouped species, with its geographical distribution; these valid taxa include more than 400 synonyms. Abbreviation: STA (Species tentatively accepted).

**SECT. ARTHRAGROSTIS**	
*Panicum aristispiculum* (B.K. Simon) Zuloaga	AUSTRALIA
*Panicum brassianum* (B.K. Simon) Zuloaga	AUSTRALIA
*Panicum brassianum* var. *minutiflorum* (B.K. Simon) Zuloaga	AUSTRALIA
*Panicum caudiglume* Hack.	ASIA
*Panicum chillagoanum* B.K. Simon	AUSTRALIA
*Panicum clarksonianum* (B.K. Simon) Zuloaga	AUSTRALIA
*Panicum deschampsioides* Domin	AUSTRALIA
*Panicum mindanaense* Merr.	ASIA
*Panicum robustum* B.K. Simon	AUSTRALIA
*Panicum seminudum* Domin	AUSTRALIA
*Panicum trachyrhachis* Benth.	AUSTRALIA
**SECT. DICHOTOMIFLORA**	
*Panicum aquaticum* Poir. var. *aquaticum*	AMERICA
*Panicum aquaticum* Poir. var. *cartagoense* Davidse	AMERICA
*Panicum bechuanense* Brem. & Ober.	AFRICA
*Panicum dichotomiflorum* Michx.	AMERICA (introduced in the Old World?)
*Panicum gilvum* Launert	AFRICA
*Panicum lacustre* Hitchc. & Ekman	AMERICA
*Panicum luzonense* J. Presl	ASIA
*Panicum mlahiense* Renvoize	AFRICA
*Panicum obseptum* Trin.	AUSTRALIA
*Panicum paludosum* Roxb. (STA)	ASIA
*Panicum perangustatum* Renvoize	AFRICA
*Panicum porphyrrhizos* Steud.	AFRICA
*Panicum psilopodium* Trin. (STA)	ASIA
*Panicum schinzii* Hack.	AFRICA
*Panicum subalbidum* Kunth	AFRICA
*Panicum sublaeve* Swallen	AMERICA
*Panicum sumatrense* Roth ex Roem. & Schult.	ASIA
*Panicum vaseyanum* Scribn. Ex Beal	AMERICA
**SECT. HIANTES**	
*Panicum afzelii* Sw.	AFRICA
*Panicum altum* Hithc. & Chase	AMERICA
*Panicum amarum* Elliott var. *amarum*	NORTH AND CENTRAL AMERICA
*Panicum amarum* var. *amarulum* (Hitchc. & Chase) Palmer	NORTH AND CENTRAL AMERICA
*Panicum anabaptistum* Steud.	AFRICA
*Panicum callosum* Hochst.	AFRICA
*Panicum chloroleucum* Griseb.	SOUTH AMERICA
*Panicum complanatum* Guglieri, Longhi-Wagner & Zuloaga	SOUTH AMERICA
*Panicum curviflorum* Hornerm.	ASIA
*Panicum deciduum* Swallen	SOUTH AMERICA
*Panicum dewinteri* J.G. Anderson	AFRICA
*Panicum fischeri* Bor	ASIA
*Panicum fluviicola* Steud.	AFRICA
*Panicum genuflexum* Stapf	AFRICA
*Panicum glabripes* Döll	SOUTH AMERICA
*Panicum glaucifolium* Hitchc. (STA)	AFRICA
*Panicum griffoni* Franch.	AFRICA
*Panicum hanningtoniii* Stapf	AFRICA
*Panicum havardii* Vasey	NORTH AMERICA
*Panicum humile* Nees ex Steud.	ASIA, AFRICA
*Panicum kalaharense* Mez	AFRICA
*Panicum kasumense* Renvoize (STA)	AFRICA
*Panicum longissimum* (Mez) Henrard	SOUTH AMERICA
*Panicum massaiense* Mez	AFRICA
*Panicum mystasipum* Zuloaga & Morrone	SOUTH AMERICA
*Panicum nigerense* Hitchc. (STA)	AFRICA
*Panicum olyroides* Kunth var. *olyroides*	SOUTH AMERICA
*Panicum olyroides* Kunth var. *hirsutum* Henrard	SOUTH AMERICA
*Panicum pansum* Rendle	AFRICA
*Panicum paucinode* Stapf	AFRICA
*Panicum phragmitoides* Stapf	AFRICA
*Panicum pilgeri* Mez	AFRICA
*Panicum pooides* Stapf	AFRICA
*Panicum racemosum* (P. Beauv.) Spreng.	SOUTH AMERICA
*Panicum ruspolii* Chiov.	AFRICA
*Panicum tricholaenoides* Steud. var. *flavomarginatum* (Mez) Zuloaga	SOUTH AMERICA
*Panicum tricholaenoides* Steud. var. *tricholaenoides*	SOUTH AMERICA
*Panicum turgidum* Forssk.	AFRICA, ASIA
*Panicum urvilleanum* Kunth	NORTH AND SOUTH AMERICA
*Panicum virgatum* L.	NORTH AMERICA
*Panicum zambesiense* Renvoize	AFRICA
**SECT. PANICUM**	
*Panicum alatum* Zuloaga & Morrone var. *alatum*	NORTH AMERICA
*Panicum alatum* var. *major* Zuloaga & Morrone	NORTH AMERICA
*Panicum alatum* var. *minor* Zuloaga & Morrone	NORTH AMERICA & SOUTH AMERICA
*Panicum aquarum* Zuloaga & Morrone	SOUTH AMERICA
*Panicum arcurameum* Stapf	AFRICA
*Panicum atrosanguineum* A. Rich.	AFRICA
*Panicum aztecanum* Zuloaga & Morrone	NORTH AMERICA
*Panicum beecheyi* Hook. & Arn.	SANDWICH IS.
*Panicum bergii* Arechav. var. *bergii*	SOUTH AMERICA
*Panicum bergii* var. *pilosissimum* Zuloaga	SOUTH AMERICA
*Panicum bombycinum* B.K. Simon	AUSTRALIA
*Panicum capillare* L.	NORTH AMERICA
*Panicum capillarioides* Vasey	NORTH AMERICA
*Panicum carneovaginatum* Renvoize	AFRICA
*Panicum carteri* Hosaka (STA)	SANDWICH IS.
*Panicum chasei* Roseng., B.R. Arrill. & Izag.	SOUTH AMERICA
*Panicum congoense* Franch.	AFRICA
*Panicum decolorans* Kunth	NORTH AND CENTRAL AMERICA
*Panicum diffusum* Sw.	CARIBBEAN
*Panicum dregeanum* Nees	AFRICA
*Panicum effusum* R. Br.	AUSTRALIA
*Panicum ephemeroides* Zuloaga & Morrone	SOUTH AMERICA
*Panicum ephemerum* Renvoize	AFRICA
*Panicum exiguum* Mez	SOUTH AMERICA
*Panicum flexile* (Gatt.) Scribn.	NORTH AMERICA
*Panicum fauriei* Hitchc.	SANDWICH IS.
*Panicum furvum* Swallen	CENTRAL AMERICA
*Panicum ghiesbreghtii* E. Fourn.	NORTH AMERICA, THE CARIBBEAN AND SOUTH AMERICA
*Panicum haplocaulos* Pilg.	AFRICA
*Panicum hippothrix* K. Schum.	AFRICA
*Panicum hallii* Vasey var. *hallii*	NORTH AMERICA
*Panicum hallii* Vasey var. *filipes* (Scribn.) F.R. Waller	NORTH AMERICA
*Panicum hillmanii* Chase	NORTH AMERICA
*Panicum hirsutum* Sw.	NORTH AMERICA, CENTRAL AMERICA, THE CARIBBEAN AND SOUTH AMERICA
*Panicum hirticaule* J. Presl var. *hirticaule*	NORTH AMERICA, CENTRAL AMERICA AND SOUTH AMERICA
*Panicum hirticaule* J. Presl var. *verrucosum* Zuloaga & Morrone	NORTH AMERICA
*Panicum hispidifolium* Swallen	CENTRAL AND SOUTH AMERICA
*Panicum konaense* Whitney & Hosaka	SANDWICH IS.
*Panicum lepidulum* Hitchc. & Chase	NORTH AND CENTRAL AMERICA
*Panicum lineale* H. St. John	SANDWICH IS.
*Panicum madipirense* Mez	AFRICA
*Panicum magnispicula* Zuloaga, Morrone & Valls	SOUTH AMERICA
*Panicum miliaceum* L.	ASIA
*Panicum mohavense* Reeder	NORTH AMERICA
*Panicum mucronulatum* Mez	SOUTH AMERICA
*Panicum nephelophilum* Gaudich.	SANDWICH IS.
*Panicum novemnerve* Stapf	AFRICA
*Panicum nubigenum* Kunth	SANDWICH IS.
*Panicum pampinosum* Hitchc. & Chase	NORTH AMERICA
*Panicum parcum* Hitchc. & Chase	NORTH AND CENTRAL AMERICA
*Panicum peladoense* Henrard	SOUTH AMERICA
*Panicum philadelphicum* Bernh. ex Trin.	NORTH AMERICA
*Panicum phoiniclados* Naik & Patunkar	ASIA
*Panicum quadriglume* (Döll) Hitchc.	SOUTH AMERICA
*Panicum ramosius* Hitchc. (STA)	SANDWICH IS.
*Panicum shinyangense* Renvoize	AFRICA
*Panicum simile* Domin	AUSTRALIA
*Panicum simulans* Smook	AFRICA
*Panicum stramineum* Hitchc. & Chase	NORTH AMERICA, CENTRAL AND SOUTH AMERICA
*Panicum tamaulipense* F.R. Waller & Morden	NORTH AMERICA
*Panicum torridum* Gaudich.	SANDWICH IS.
*Panicum venosum* Swallen	NORTH AMERICA
*Panicum volutans* J.G. Anderson	AFRICA
*Panicum xerophilum* (Hillebr.) Hitchc.	SANDWICH IS.
**SECT. REPENTIA**	
*Panicum arbusculum* Mez	AFRICA
*Panicum assumptionis* Stapf	MASCARENES
*Panicum buncei* F. Muell. ex Benth.	ASIA (AUSTRALIA)
*Panicum coloratum* L.	AFRICA
*Panicum decompositum* R. Br.	AUSTRALIA
*Panicum gouinii* E. Fourn.	AMERICA
*Panicum hygrocharis* Steud.	AFRICA
*Panicum joshuae* Lambdon (STA)	ST. HELENA
*Panicum laevinode* Lindl.	AUSTRALIA
*Panicum lanipes* Mez	AFRICA
*Panicum larcomianum* Mez	AUSTRALIA
*Panicum latzii* R.Webster	AUSTRALIA
*Panicum merkeri* Mez	AFRICA
*Panicum pedersenii* Zuloaga	AMERICA
*Panicum pinifolium* Chiov.	AFRICA
*Panicum repens* L.	AFRICA
*Panicum rigidum* Balfour	SOCOTRA
*Panicum socotranum* Cope (STA)	SOCOTRA
*Panicum stapfianum* Fourc.	AFRICA
*Panicum subflabellatum* Stapf	AFRICA
**SECT. RUDGEANA**	
*Panicum cayennense* Lam.	CENTRAL AND SOUTH AMERICA, CARIBBEAN
*Panicum campestre* Nees ex Trin.	SOUTH AMERICA
*Panicum cervicatum* Chase	SOUTH AMERICA
*Panicum ligulare* Nees ex Trin.	SOUTH AMERICA
*Panicum rudgei* Roem. & Schult.	CENTRAL AND SOUTH AMERICA, CARIBBEAN
**SECT. YAKIRRA**	
*Panicum australiense* Domin var. *australiense*	AUSTRALIA
*Panicum australiense* var. *intermedium* (R.D. Webster) Zuloaga	AUSTRALIA
*Panicum foliolosum* (Munro ex Hook. f.) Stieber	AUSTRALIA
*Panicum majusculum* F. Muell. ex Benth.	AUSTRALIA
*Panicum muelleri* (Hughes) Lazarides	AUSTRALIA
*Panicum nullum* (Lazarides & R.D. Webster) Zuloaga	AUSTRALIA
*Panicum pauciflorum* R. Br.	AUSTRALIA
*Panicum websterii* (B.K. Simon) Zuloaga	AUSTRALIA
**UNGROUPED SPECIES**	
*Panicum cinctum* Hack.	MADAGASCAR
*Panicum laetum* Kunth	AFRICA
*Panicum luridum* Hack. ex S. Elliott	MADAGASCAR
*Panicum mitchelii* Benth.	AUSTRALIA
*Panicum pilgerianum* (Schweick.) Clayton	AFRICA
*Panicum queenslandicum* Domin	AUSTRALIA
*Panicum voeltzkowii* Mez	MADAGASCAR

**Table 5:** List of species to be excluded from *Panicum*. Ph-p. refers to photosynthetic pathway; U: Unknown.

Species	Distribution	Taxonomic placement	Ph-p.
*Panicum acrotrichum* Hook. f.	AFRICA	INCERTAE SEDIS GENUS	C_3_
*Panicum aequinerve* Nees	AFRICA	INCERTAE SEDIS GENUS	C_3_
*Panicum ambositrense* A. Camus	MADAGASCAR	INCERTAE SEDIS GENUS	C_3_
*Panicum amoenum* Balansa	ASIA	INCERTAE SEDIS GENUS	C_3_
*Panicum andrigintrense* A. Camus	MADAGASCAR	BOIVINELLINAE	C_3_
*Panicum ankarense* A. Camus	MADAGASCAR	INCERTAE SEDIS GENUS	C_3_
*Panicum antidotale* Retz.	ASIA	CENCHRINAE	C_4_
*Panicum bambusiculme* Friis & Vollesen	AFRICA	INCERTAE SEDIS GENUS	C_3_
*Panicum bartlettii* Swallen	CENTRAL AMERICA	INCERTAE SEDIS GENUS	C_3_
*Panicum bisulcatum* Thunb.	ASIA	SACCIOLEPIS?	C_3_
*Panicum bresolinii* L.B. Sm. & Wassh.	SOUTH AMERICA	OTACHYRIINAE	C_3_
*Panicum brevifolium* L.	AFRICA	INCERTAE SEDIS GENUS	C_3_
*Panicum calocarpum* Berhaut	AFRICA	INCERTAE SEDIS GENUS	U
*Panicum calvum* Stapf	AFRICA	INCERTAE SEDIS GENUS	C_3_
*Panicum capuronii* A. Camus	MADAGASCAR	INCERTAE SEDIS GENUS	C_3_
*Panicum chambeshii* Renvoize	AFRICA	INCERTAE SEDIS GENUS	C_3_
*Panicum chionachne* Mez	AFRICA	INCERTAE SEDIS GENUS	C_3_
*Panicum chusqueoides* Hack.	AFRICA	INCERTAE SEDIS GENUS	U
*Panicum comorense* Mez	AFRICA	BOIVINELLINAE	C_3_
*Panicum condensatum* Raddi	SOUTH AMERICA	OTACHYRIINAE	C_3_
*Panicum crystallinum* Judz. & Voronts.	MADAGASCAR	INCERTAE SEDIS GENUS	U
*Panicum cupressifolium* A. Camus	MADAGASCAR	INCERTAE SEDIS GENUS	C_3_
*Panicum danguyi* A. Camus	MADAGASCAR	INCERTAE SEDIS GENUS	U
*Panicum delicatulum* Fig. & De Not.	AFRICA	INCERTAE SEDIS GENUS	C_3_
*Panicum deustum* Thunb.	AFRICA	MELINIDINAE	C_4_
*Panicum dorsense* S.M. Phillips	AFRICA	INCERTAE SEDIS GENUS	C_3_
*Panicum eickii* Mez	AFRICA	INCERTAE SEDIS GENUS	C_3_
*Panicum flacourtii* A. Camus	MADAGASCAR	INCERTAE SEDIS GENUS	C_3_
*Panicum gardneri* Thw.	ASIA	INCERTAE SEDIS GENUS	C_3_
*Panicum glandulopaniculatum* Renvoize	AFRICA	INCERTAE SEDIS GENUS	C_3_
*Panicum haenkeanum* J. Presl	CENTRAL AND SOUTH AMERICA	INCERTAE SEDIS GENUS	C_3_
*Panicum harleyi* Salariato, Morrone & Zuloaga	SOUTH AMERICA	OTACHYRIINAE	C_3_
*Panicum hayatae* A. Camus	ASIA	INCERTAE SEDIS GENUS	C_3_
*Panicum heterostachyum* Hack. (STA)	AFRICA	INCERTAE SEDIS GENUS	C_3_
*Panicum hirtum* Lam.	SOUTH AMERICA	INCERTAE SEDIS GENUS	C_3_
*Panicum hochstetteri* Steud.	AFRICA	INCERTAE SEDIS GENUS	C_3_
*Panicum humbertii* A. Camus	MADAGASCAR	INCERTAE SEDIS GENUS	C_3_
*Panicum humidorum* Buch.-Ham. ex Hook. f.	ASIA	INCERTAE SEDIS GENUS	C_3_
*Panicum ibityense* A. Camus	MADAGASCAR	INCERTAE SEDIS GENUS	C_3_
*Panicum inaequilatum* Stapf & Hubb.		INCERTAE SEDIS GENUS	C_3_
*Panicum incisum* Munro	ASIA	INCERTAE SEDIS GENUS	C_3_
*Panicum inconspicuum* Voronts.	MADAGASCAR	INCERTAE SEDIS GENUS	U
*Panicum isolepis* Mez	AFRICA	INCERTAE SEDIS GENUS	C_3_
*Panicum issongense* Pilger	AFRICA	INCERTAE SEDIS GENUS	C_3_
*Panicum khasianum* Munro ex Hook. f.	ASIA (INDIA)	INCERTAE SEDIS GENUS	C_3_
*Panicum lachnophyllum* Benth.	AUSTRALIA	INCERTAE SEDIS GENUS	C_3_
*Panicum laticomum* Nees	AFRICA	INCERTAE SEDIS GENUS	C_3_
*Panicum letouzeyi* Renvoize	AFRICA	INCERTAE SEDIS GENUS	U
*Panicum longipedicellatum* Swallen	SOUTH AMERICA	INCERTAE SEDIS GENUS	C_3_
*Panicum longum* Hitchc. & Chase	CENTRAL AMERICA	OTACHYRIINAE	C_3_
*Panicum manongarivense* A. Camus	MADAGASCAR	INCERTAE SEDIS GENUS	C_3_
*Panicum mapalense* Pilg.	AFRICA	INCERTAE SEDIS GENUS	C_3_
*Panicum millegrana* Poir.	CENTRAL AND SOUTH AMERICA	INCERTAE SEDIS GENUS	C_3_
*Panicum mitopus* K. Schum.	AFRICA	INCERTAE SEDIS GENUS	C_3_
*Panicum monticola* Hook. f.	AFRICA	INCERTAE SEDIS GENUS	C_3_
*Panicum notatum* Retz.	ASIA	INCERTAE SEDIS GENUS	C_3_
*Panicum nudiflorum* Renvoize	AFRICA	INCERTAE SEDIS GENUS	U
*Panicum nymphoides* Renvoize	AFRICA	INCERTAE SEDIS GENUS	C_3_
*Panicum obumbratum* Stapf	AFRICA	INCERTAE SEDIS GENUS	C_3_
*Panicum paianum* Naik & Patunkar	ASIA	INCERTAE SEDIS GENUS	U
*Panicum palackyanum* A. Camus	MADAGASCAR	INCERTAE SEDIS GENUS	C_3_
*Panicum peregrinum* Steud.	AFRICA	ADENOCHLOA	C_3_
*Panicum perrieri* A. Camus	MADAGASCAR	INCERTAE SEDIS GENUS	C_3_
*Panicum phipsii* Renvoize	AFRICA	INCERTAE SEDIS GENUS	C_3_
*Panicum pleianthum* Peter	AFRICA	INCERTAE SEDIS GENUS	C_3_
*Panicum pseudoracemosum* Renvoize	AFRICA	INCERTAE SEDIS GENUS	C_3_
*Panicum pusillum* Hook. f.	AFRICA	INCERTAE SEDIS GENUS	C_3_
*Panicum pygmaeum* R. Br.	ASIA (AUSTRALIA)	BOIVINELLINAE	C_3_
*Panicum robynsii* A. Camus	AFRICA	BOIVINELLINAE	C_3_
*Panicum sabiense* Renvoize	AFRICA	MEGATHYRSUS?	C_4_
*Panicum saigonense* Mez	ASIA	HYMENACHNE?	C_3_
*Panicum sarmentosum* Roxb.	ASIA	INCERTAE SEDIS GENUS	C_3_
*Panicum sellowii* Nees	CENTRAL AND SOUTH AMERICA	INCERTAE SEDIS GENUS	C_3_
*Panicum smithii* M.M. Rhaman	ASIA	INCERTAE SEDIS GENUS	U
*Panicum spergulifolium* A. Camus	AFRICA	INCERTAE SEDIS GENUS	C_3_
*Panicum spongiosum* Stapf	AFRICA	INCEERTAE SEDIS GENUS	C_3_
*Panicum striatissimum* C.E. Hubb.	AFRICA	INCERTAE SEDIS GENUS	C_3_
*Panicum subhystrix* A. Camus	MADAGASCAR	INCERTAE SEDIS GENUS	C_3_
*Panicum trichanthum* Nees	CENTRAL AMERICA, THE CARIBBEAN AND SOUTH AMERICA	INCERTAE SEDIS GENUS	C_3_
*Panicum trichocladum* Hack. ex K. Schum.	AFRICA	MELINIDINAE	C_4_
*Panicum trichoides* Sw.	CENTRAL AMERICA, THE CARIBBEAN AND SOUTH AMERICA	INCERTAE SEDIS GENUS	C_3_
*Panicum vohitrense* A. Camus	MADAGASCAR	INCERTAE SEDIS GENUS	C_3_
*Panicum vollesenii* Renvoize	AFRICA	INCERTAE SEDIS GENUS	U

1. **Panicum** sect. **Arthragrostis** (Lazarides) Zuloaga, **comb. nov**. *Arthragrostis* Lazarides, Nuytsia 5(2): 285. 1984. Type species: *Panicum deschampsioides* Domin, Biblioth. Bot. 20(85): 320. 1915.

**Fig 5:**
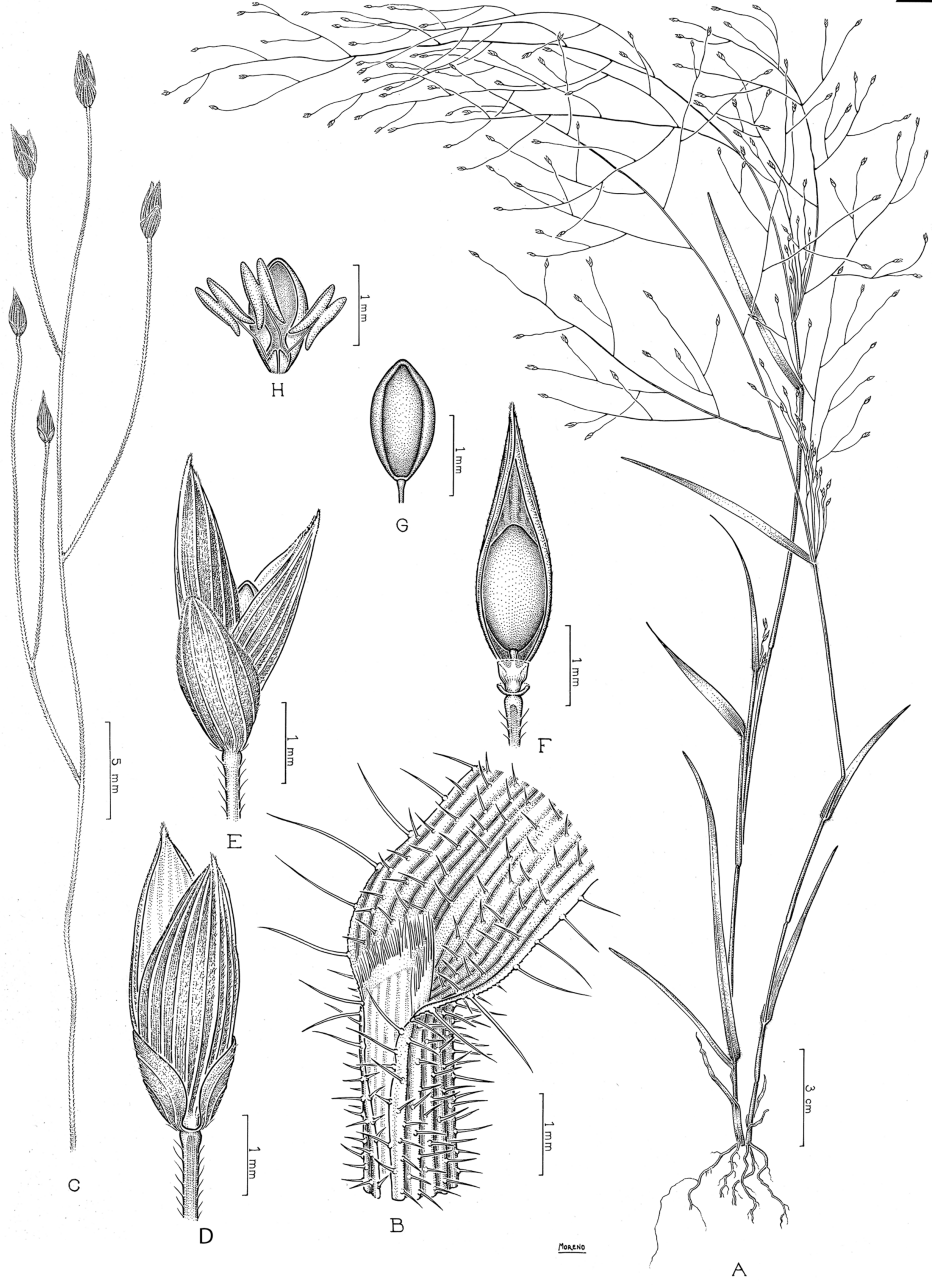
Sect. Arthragrostis. *Panicum deschampsioides*. A. Habit. B. Detail of ligule. C. Detail of the inflorescence. D. Spikelet, upper glume view. E. Spikelet, lateral view. F. Upper anthecium, dorsal view, and lower lemma. G. Upper anthecium, palea view. H. Upper palea with lodicules and stamens.

Annual or perennials, erect to decumbent and rooting at the lower nodes; internodes hollow, glabrous. *Ligules* membranous-ciliate. *Blades* lanceolate, flat, pilose, the margins usually ciliate. *Inflorescence* an open and lax panicle. *Spikelets* ovoid to narrowly ovoid or ellipsoid, glabrous, less frequently covered by tuberculate hairs; lower glume less than ½ the length of the spikelet, separated by a distinct internode from the upper glume, 5-7-nerved; upper glume and lower lemma subequal, awned to acuminate or acute, 7-11-nerved, membranous, with a manifest rhachilla between the bracts; lower palea reduced, lower flower absent; *upper anthecium* shorter than the upper glume and lower lemma, stipitate or not, indurate, pale to dark, glabrous. (Fig 5).

Including ten Old World species, four of which require combinations in *Panicum*. [107] classified the genus *Arthragrostis* as endemic to Australia. Nevertheless, two species are present in the Philippines, *P*. *caudiglume* Hack. and *P*. *mindanaense* Merr., the former also growing in Java.

***Panicum aristispiculum*** (B.K. Simon) Zuloaga, **comb. nov**. *Arthragrostis aristispicula* B.K. Simon, Austrobaileya 2(3): 238. 1986. Type: Australia. Queensland: Brisbane District: Almaden-Petford road, 4 km from Almaden, 10 Mar, 1980, *B*. *K*. *Simon* & *J*. *R*. *Clarkson 3598* (holotype, BRI!).
***Panicum brassianum*** (B.K. Simon) Zuloaga, **comb. nov**. *Arthragrostis brassiana* B.K. Simon, Austrobaileya 8(2): 188. 2010. Type: Australia. Queensland: Cook District: crest of Western Scarp of Great Dividing Range, 12 mi E of The Lynd, 11 July 1954, *S*.*T*. *Blake 19478* (holotype, BRI!; isotypes, AD, CANB!, DNA, K!, L, MO!, PERTH, PRE).
***Panicum brassianum*** var. **minutiflorum** (B.K. Simon) Zuloaga, **comb. nov**. *Arthragostis brassiana* var. *minutiflora* B.K. Simon, Austrobaileya 8(2): 188. 2000. Type: Australia. Queensland: Cook District: Lockerbie, 10 mi W of Somerset, 4 May 1948, *L*.*J*. *Brass 18637* (holotype, BRI!; isotype, A).
***Panicum clarksonianum*** (B.K. Simon) Zuloaga, **comb. nov**. *Arthragrostis clarksoniana* B.K. Simon, Austrobaileya 3(4): 585. 1992. Type: Australia. Cook District: 16 km from Meripah homestead on road to the south, 13°49'S, 142°22'E, 11 May 1987, *J*.*R*. *Clarkson 7149* & *B*.*K*. *Simon* (holotype, BRI!; isotypes, CNS!, MBA, NSW!).
2. **Panicum** sect. **Dichotomiflora** (Hitchc.) Hitchc. & Chase ex Honda, J. Fac. Sci. Univ. Tokyo, Sect. 3, Bot. 3(1): 244, 246. 1930. *Panicum* [unranked] *Dichotomiflora* Hitchc., N. Amer. Fl. 3(2): 200, 202. 1915. *Panicum* group *Dichotomiflora* Hitchc. & Chase, Contr. U.S. Natl. Herb. 15: 28, 47. 1910, nom. inval. Type species: *Panicum dichotomiflorum* Michx.

**Fig 6:**
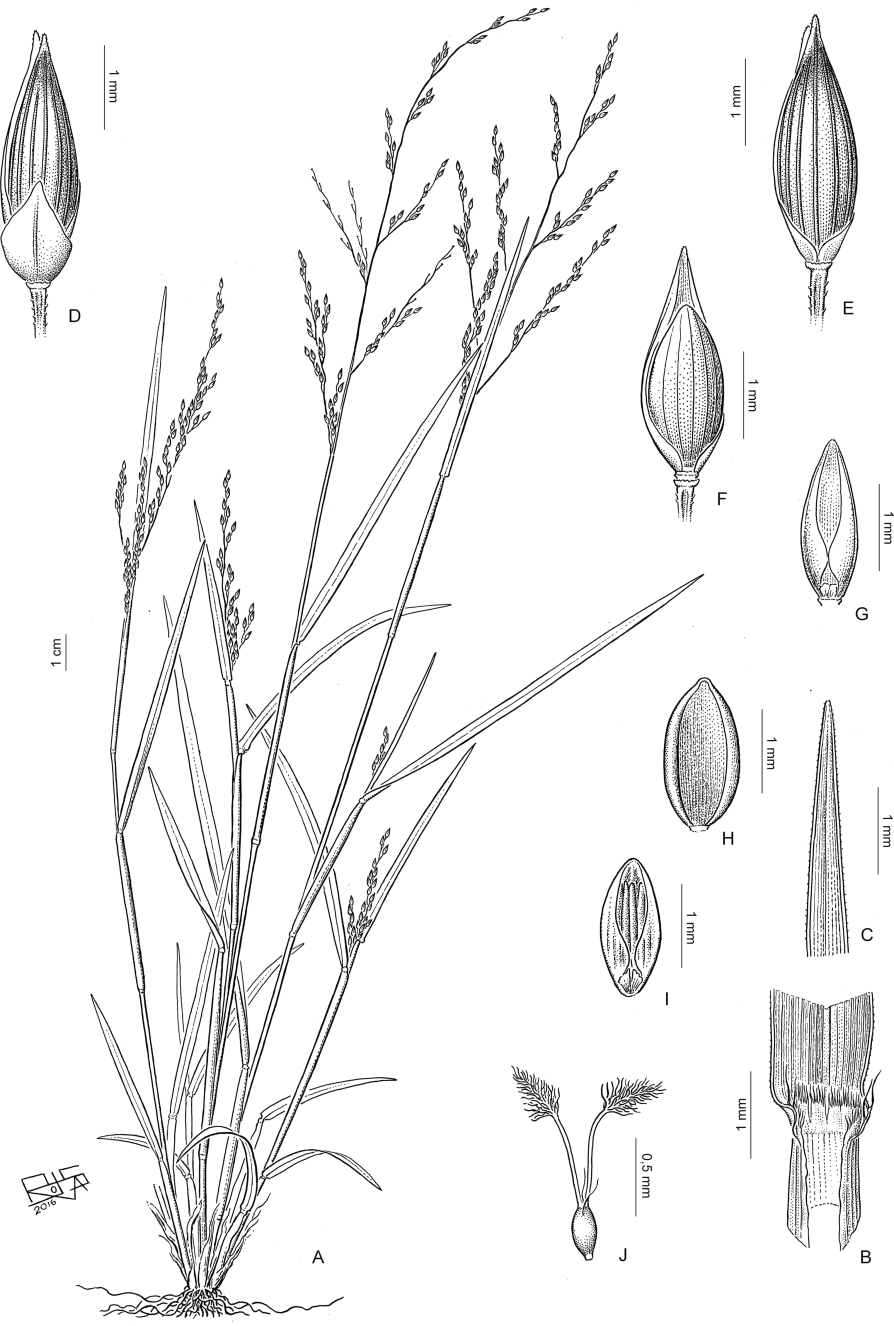
Sect. Dichotomiflora. *Panicum impeditum*. A. Habit. B. Detail of ligule. C. Apex of blade. D. Spikelet, lower glume view. E. Spìkelet, upper glume view. F. Upper anthecium and lower lemma. G. Lower palea, ventral view. H. Upper anthecium, palea view. I. Upper palea with lodicules and anthers. J. Gynoecium.

Annual, occasionally perennials, with culms erect to decumbent, rooting and branching at the lower nodes. *Blades* lanceolate. *Inflorescence* a terminal and open, diffuse to contracted, panicle. *Spikelets* ellipsoid to lanceolate, glabrous; lower glume 1/5 to 1/3 the length of the spikelet, 1-3-nerved; upper glume and lower lemma subequal, 5-7(-9) nerved; upper anthecium smooth, indurate. (Fig 6).

This section includes four species in America, approximately twelve in the Old World, with *P*. *dichotomiflorum* widely distributed worldwide. They are frequent in humid and wet, open areas, usually present in river banks.

3. **Panicum** sect. **Hiantes** Stapf, Fl. Trop. Afr. 9: 640, 644. 1920. Type species: *P*. *phragmitoides* Stapf.
*Panicum* sect *Durae* Stapf, Fl. Trop. Afr. 9: 640, 648. 1920. Type species: *Panicum turgidum* Forssk., lectotype here designated.
*Panicum* sect. *Urvilleana* (Hitchc.) Pilger, Notizbl. Bot. Gart. Berlin-Dahlem 11(104): 244. 1931. *Panicum* group *Urvilleana* Hitchc. & Chase, Contr. U.S. Natl. Herb. 15: 28, 132. 1910, nom. inval. *Panicum* [unranked] *Urvilleana* Hitchc., N. Amer. Fl. 3(2): 200, 205. 1915.
*Panicum* sect. *Virgata* Hitchc. & Chase ex Pilg., Nat. Pflanzenfam. (ed. 2), 14e: 22. 1940. *Panicum* group *Virgata* Hitchc. & Chase, Contr. U.S. Natl. Herb. 15: 29, 84. 1910, nom. inval. *Panicum* [unranked] *Virgata* Hitchc., N. Amer. Fl. 3(2): 200, 203. 1915.

**Fig 7:**
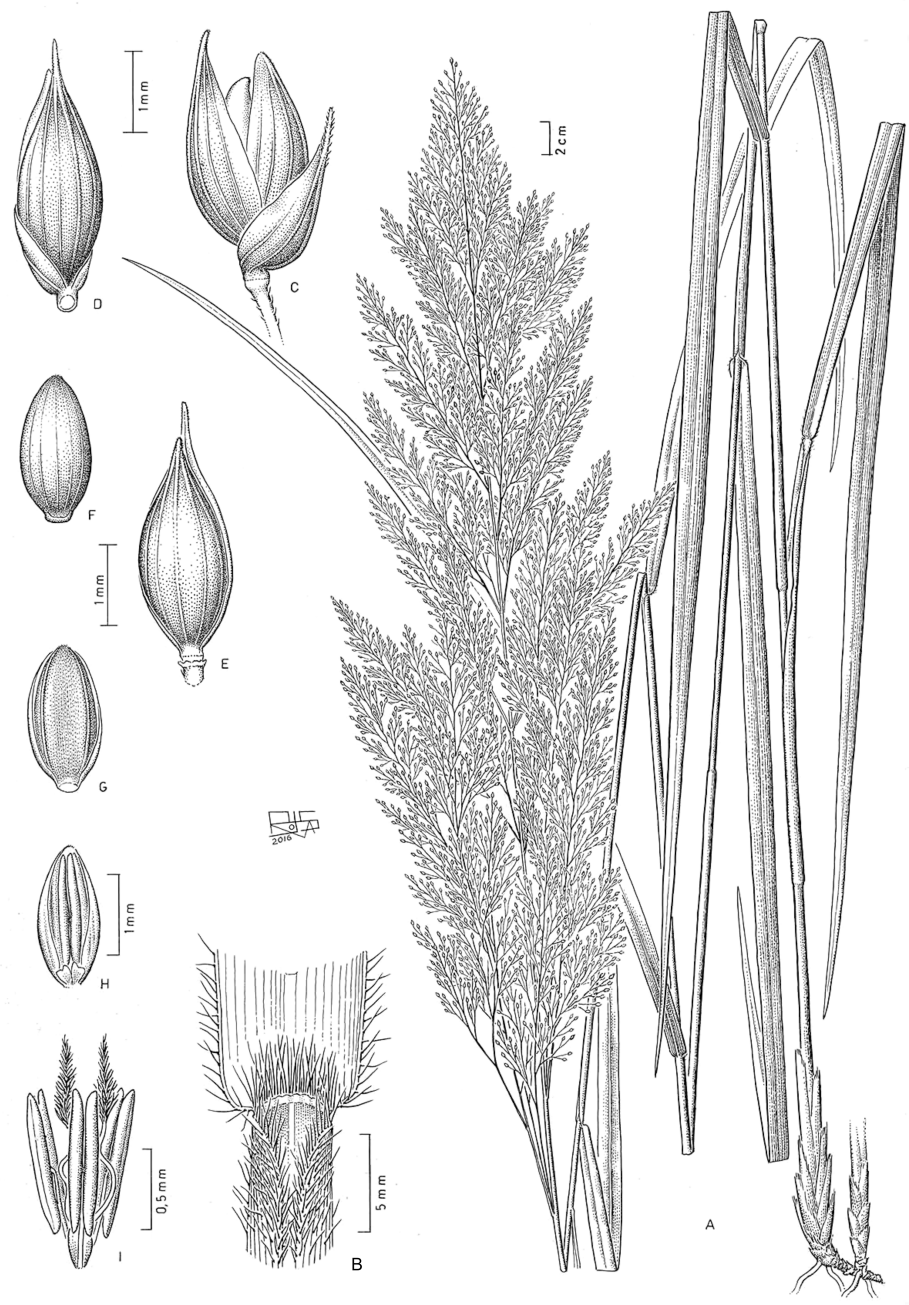
Sect. Hiantes. *Panicum phragmitoides*. A. Habit. B. Detail of ligule. C. Spikelet, lateral view. D. Spikelet, upper glume view. E. Upper anthecium and lower lemma. F. Upper anthecium, lemma view. G. Upper anthecium, palea view. H. Upper palea with lodicules and anthers. I. Lodicules, stamens and gynoecium.

Annual or cespitose perennials, culms simple, erect or geniculate ascending. *Blades* linear to lanceolate, flat or involute. *Inflorescence* an open, oblong to ovate panicle. Spikelets gaping at maturity, silky villous to pilose or glabrous; lower glume ¾ to the full length of the spikelet, upper glume and lower lemma subequal, longer than the upper anthecium, (5-)7-9(-11) nerved; lower palea conspicuous and lower flower male; upper anthecium indurate, smooth, shiny. (Fig 7).

The section includes fourteen perennial American species, and nearly 24 species in the Old World, five of them annual; they are usually found in open and dry or mesophytic environments.

4. **Panicum** sect. **Panicum**

*Panicum* sect. *Capillare* (Hitchc.) Fernald, Rhodora 21(246): 110. 1919. *Panicum* [unranked] *Capillaria* Hitchc., N. Amer. Fl. 3(2): 200, 206. 1915. *Panicum* group *Capillaria* Hitchc. & Chase, Contr. U.S. Natl. Herb. 15: 28, 54. 1910, nom. inval.
*Panicum* group *Diffusa* Hitchc., Contr. U.S. Natl. Herb. 15: 29, 71. 1910, nom. inval. *Panicum* [unranked] *Diffusa* Hitchc., N. Amer. Fl. 17(3): 200, 203. 1915.
*Panicum* sect. *Miliaceae* Stapf, Fl. Trop. Afr. 9(4): 640, 646. 1920.

**Fig 8:**
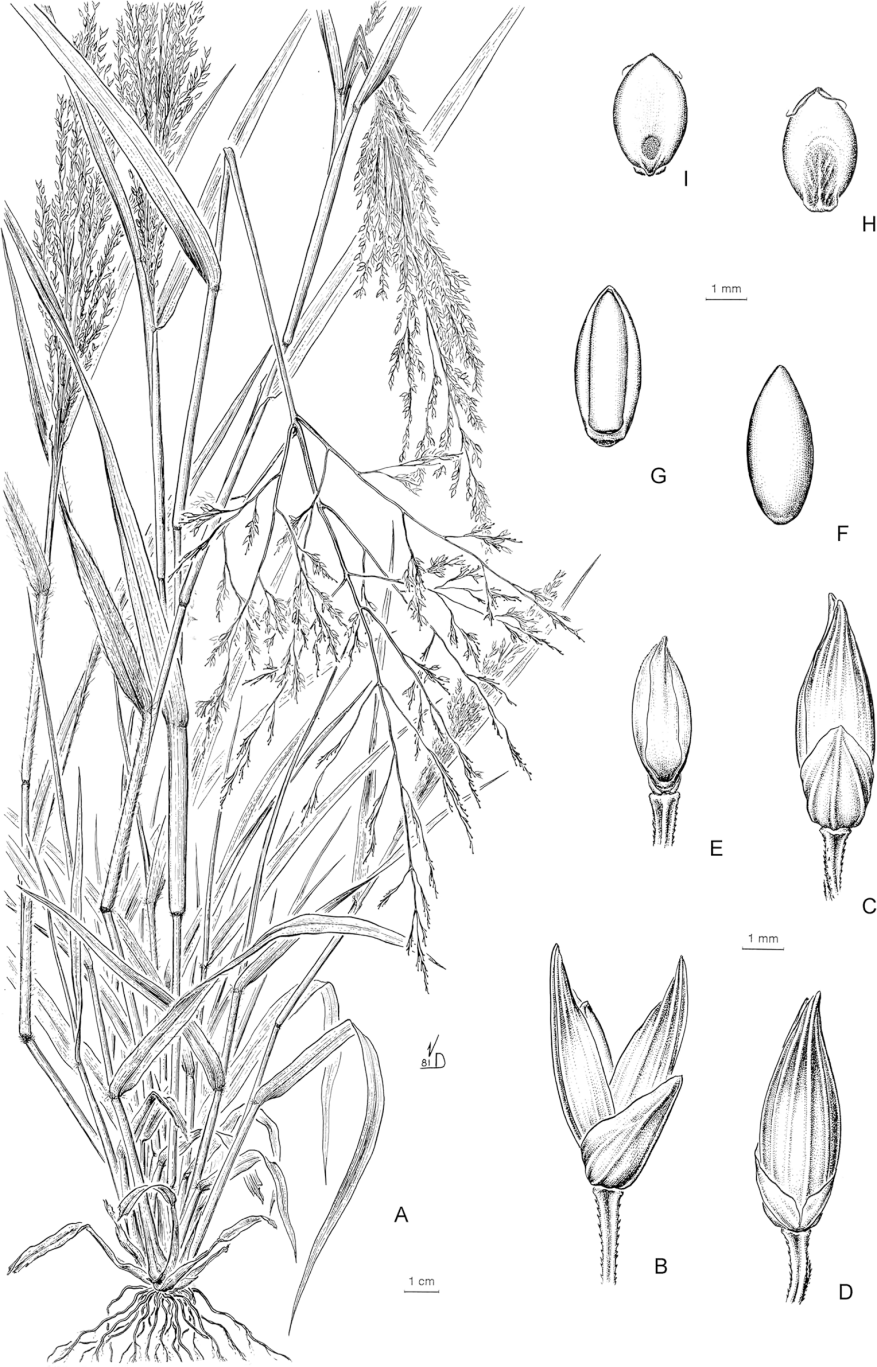
Panicum stramineum. A. Habit. B. Spikelet, lateral view. C. Spikelet, lower glume view. D. Spìkelet, upper glume view. E. Lower palea. F. Upper anthecium, dorsal view. G. Upper anthecium, palea view. H. Caryopsis, embryo view. I. Caryopsis, hilum view.

Annual or cespitose perennials, with culms erect, occasionally decumbent. *Blades* oblong-lanceolate to filiform, flat or with involute margins. *Inflorescence* an open and lax terminal panicle, axillary panicles occasionally present. *Spikelets* ovoid to long-ellipsoid, glabrous or pilose, lower glume (1/3-)1/2-3/4(-4/5) the length of the spikelet, 3-5(-9) nerved, upper glume and lower lemma (5-)7-9(-15) nerved; lower palea present or reduced to absent; lower flower male or absent; upper anthecium indurate, smooth, shiny, with simple or compound papillae toward the apex or simple papillae all over its surface. (Fig 8).

The section includes 30 American species, and ca. 28 growing in Africa, India, Asia, islands of the Pacific and Australia. They are most commonly found in dry and open areas.

*Panicum venosum* Swallen, a species transferred to the genus *Urochloa* [108], belongs to sect. *Panicum* and is strongly related in this analysis to *P*. *alatum*.

5. **Panicum** sect. **Repentia** Stapf, Fl. Trop. Afr. 9: 640, 648. 1920. Type species: *Panicum repens* L.
*Panicum* sect. *Coloratae* Stapf, Fl. Trop. Afr. 9: 641, 648. 1920.

**Fig 9:**
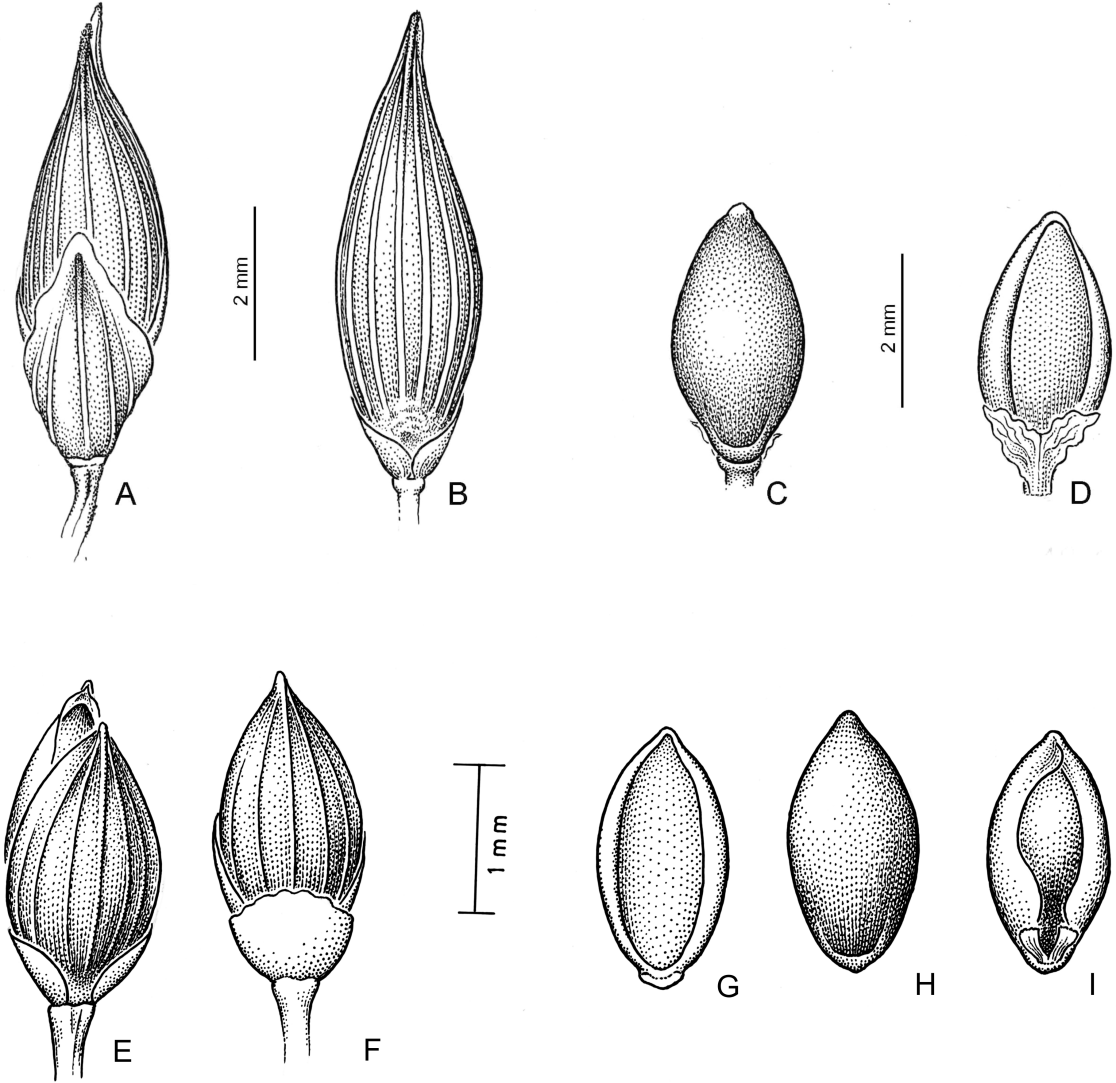
Sect. Yakirra. *Panicum majusculum*. A. Spikelet, lower glume view. B. Spikelet, upper glume view. C. Upper anthecium, palea view with stipe. D. Upper anthecium, lemma view. Sect. *Repentia*. *Panicum repens*. E. Spikelet, upper glume view. F. Spikelet, lower glume view. G. Upper anthecium, palea view. H. Upper anthecium, lemma view. I. Upper palea and lodicules.

Perennials, occasionally annuals, with stout rootstocks, culms erect. *Blades* oblong-lanceolate to lanceolate, flat to involute. *Inflorescence* a terminal, open to contracted panicle. *Spikelets* long-ovoid to ellipsoid, glabrous; lower glume ¼ to 1/3(-1/2) the length of the spikelet, 1-5(-7) nerved, upper glume and lower lemma 9–11 nerved; lower palea present, lower flower male or absent; upper anthecium indurate, smooth and shiny. (Fig 9E–9H).

The section includes two species of America (*P*. *coloratum* and *P*. *repens* introduced), and 18 species growing in the Old World, that inhabit mesophytic environments.

6. **Panicum** sect. **Rudgeana** (Hitchc.) Zuloaga, Ann. Missouri Bot. Gard. 74: 470. 1987. Type species: *Panicum rudgei* Roem. & Schult.
*Panicum* [unranked] *Rudgeana* Hitchc., N. Amer. Fl. 17(3): 200, 205. 1915.

**Fig 10:**
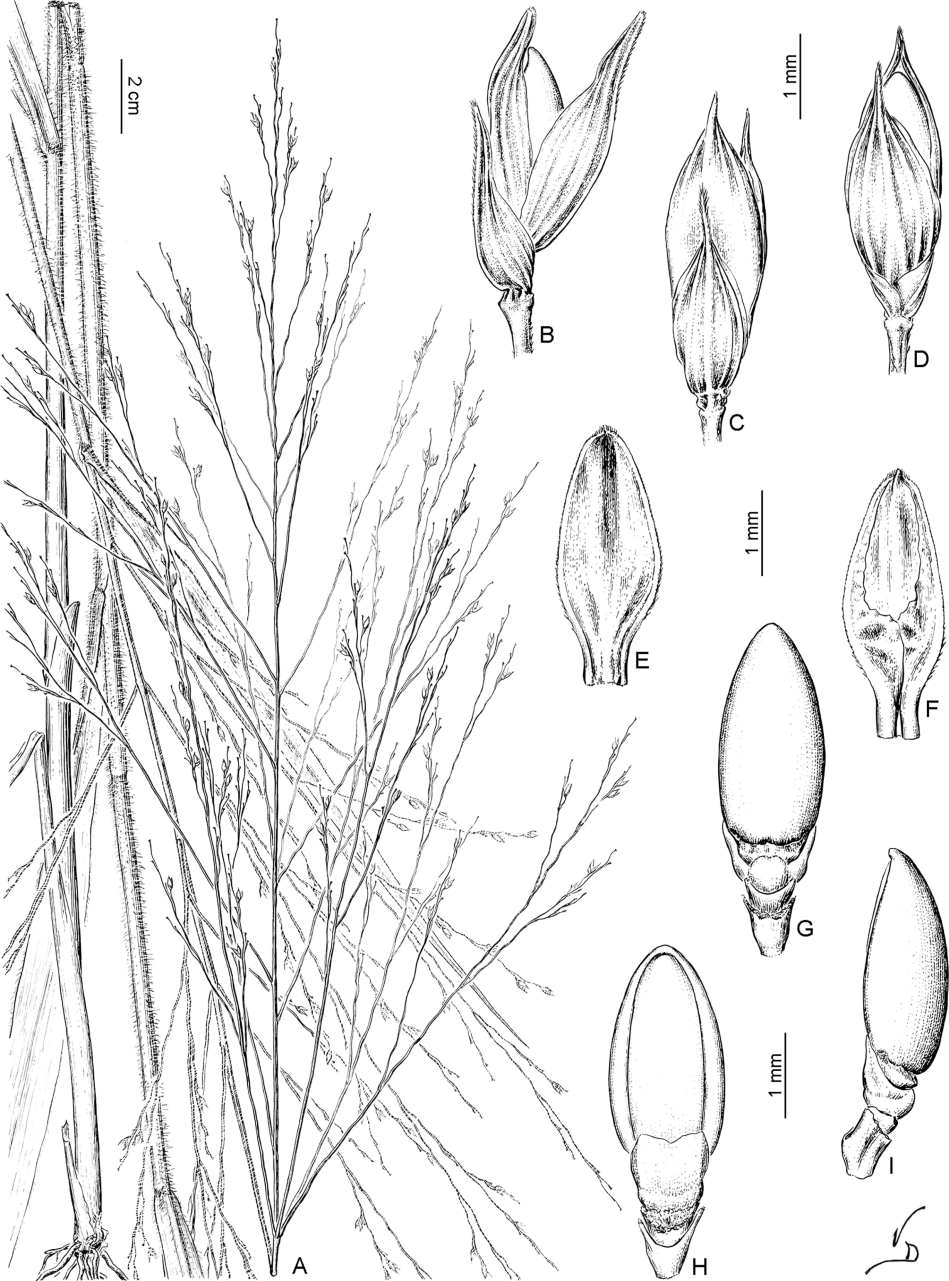
Sect. Rudgeana. *Panicum ligulare*. A. Habit. B. Spikelet, lateral view. C. Spikelet, lower glume view. D. Spikelet, upper glume view. E. Lower palea, dorsal view. F. Lower palea, ventral view. G. Upper anthecium dorsal view, with stipe. H. Upper anthecium, ventral view. I. Upper anthecium, lateral view.

Annual or cespitose perennials, with erect culms. *Blades* lanceolate to linear-lanceolate. *Inflorescence* a single, terminal and lax panicle, axillary inflorescences occasionally present. *Spikelets* obovoid to ellipsoid, pilose to glabrous, upper glume and lower lemma subequal, 5-9(-11) nerved; lower palea present, lower flower male or absent; upper anthecium stipitate, indurate, smooth, and with compound papillae at the apex. (Fig 10).

The section includes five species, two, *P*. *cayennense* Lam. and *P*. *rudgei* Roem. & Schult., widespread from Central to South America, and *P*. *cervicatum*, *P*. *ligulare* and *P*. *campestre* Nees ex Trin. growing in savannas of South America, ranging from sea-level up to near 1500 m elevation.

7. **Panicum** sect. **Yakirra** (Lazarides & R.D. Webster) Zuloaga, **comb. nov**. *Yakirra* Lazarides & R.D. Webster, Brunonia 7(2): 292. 1985. Type species: *Panicum pauciflorum* R.Br. [= *Yakirra pauciflora* (R. Br.) Lazarides & R.D. Webster]

Annual or perennial, the culms ascending or erect, branching at the lower nodes; internodes hollow, glabrous. *Ligules* membranous-ciliate. *Blades* linear to lanceolate, flat. *Inflorescence* an open, usually diffuse, panicle. *Spikelets* glabrous, with the rachilla manifest between the bracts and below the stipitate upper anthecium; lower glume 3-5-nerved, ½ or more the length of the spikelet; upper glume and lower lemma subequal, 7-9-nerved; lower palea reduced and lower flower absent; *upper anthecium* indurate, smooth, glabrous. (Fig 9A–9D).

The section includes seven species and one variety, mostly growing in open and dry areas of Australia (with one species, *P*. *foliolosum*, also present in Myanmar). Combinations for three taxa not previously named in *Panicum* are made below.

***Panicum australiense*** Domin var. ***intermedium*** (R.D. Webster) Zuloaga, **comb. nov**. *Yakirra australiense* (Domin) Lazarides & R.D. Webster var. *intermedia* R.D. Webster, Austral. Paniceae: 266. 1987. Type: Australia. Western Australia, near Lucky Hill, 23 km NNE of Dunham River, 13 Mar 1978, *M*. *Lazarides 8547* (holotype, CANB!; isotype, PERTH!).
***Panicum nullum*** (Lazarides & R.D. Webster) Zuloaga, **comb. nov**. *Yakirra nulla* Lazarides & R.D. Webster, Brunonia 7(2): 295. 1985. Type: Australia. Northern Territory: Darwin & Gulf District: 8 miles NE of Adelaide River township, 17 Mar 1965, *M*. *Lazarides* & *E*. *D*. *Adams 262* (holotype, CANB!; isotypes, DNA!, K!, L!, NT!).
***Panicum websterii*** (B.K. Simon) Zuloaga, **comb. nov**. *Yakirra websteri* B.K. Simon, Austrobaileya 3(4): 602, Fig 9. 1992. Type: Australia. Queensland: Mitchell District: 93 km N of Langlo Crossing, 1 Jul 1975, *G*. *R*. *Beeston 1361C* (holotype, BRI-AQ 268164!; isotypes, BRI!, CANB!, K!, NSW!).

## Supporting information

S1 Appendix List of taxa of the molecular analysis and GenBank accession numbers. New sequences are denoted by * and the voucher information is given (DOCX).
(DOCX)

S1 Table Areas and dispersal probabilities used in BioGeoBEARS analyses of subtribe Panicinae. Area names in rows and columns are: A, North America; B Central and South America; C, Eurasia + Mediterran + North Africa; D, Tropical and South Africa; E, Southern Asia; F, Australia. For dispersal events the ancestral areas (where the lineage dispersed from) are given in the row, and the descendent areas (where the lineage dispersed to) are given in the column. (DOCX)
(DOCX)

S2 Table Number of biogeographical events estimated in the history of the subtribe Panicinae using biogeographical stochastic mapping. Event counts were averaged across 1000 BSMs and are presented here with the standard deviations in parentheses. Total event counts are given for range-expansion events (anagenetic dispersal) (a), founder events (cladogenetic dispersal) (b), and Sympatry speciation events (c). For dispersal events the ancestral areas (where the lineage dispersed from) are given in the row, and the descendent areas (where the lineage dispersed to) are given in the column. The percentages of events involving each area either as a source (the rows) or as the destination (the columns), are given on the margins. Area names in rows and columns are: A, North America; B Central and South America; C, Eurasia + Mediterran + North Africa; D, Tropical and South Africa; E, Southern Asia; F, Australia. (DOCX)
(DOCX)

S1 Fig Maximum clade credibility (MCC) tree of Panicoideae obtained from BEAST analyses with the *ndhF* sequences, using the uncorrelated lognormal relaxed clock model and secondary calibrations based on external angiosperm fossils together with the phytolith microfossils of Poaceae (calibration scheme 2). Red boxes indicate phylogenetic placement of *Panicum* species recovered outside subtribe Panicinae. Posterior probability ≥ 0.9 are shown on the branches and horizontal bars on the nodes indicate the 95% HPD of ages. Subtribe Panicinae are shown in detail in S2 Fig Mya, million years ago; Pli, Pliocene; Plei, Pleistocene. Results from divergence time estimation based only on external angiosperm fossils calibration are shown in Fig 1. (PDF)
(PDF)

S2 Fig Divergence time estimations for subtribe Panicinae. Maximum clade credibility (MCC) tree of Panicoideae obtained from BEAST analyses with *ndhF* sequences using the uncorrelated lognormal relaxed clock model and secondary calibrations based on external angiosperm fossils together with the phytolith microfossils of Poaceae (calibration scheme 2, see materials and methods). Only subtribe Paniceae is shown in detail; for the remaining clades see S1 Fig Posterior probabilities ≥ 0.9 are shown on the branches and horizontal bars on the nodes indicate the 95% HPD of ages. Vertical bars indicate sections within *Panicum*. Paniceae 1 refers to tribe Paniceae excluding subtribes Cenchrinae, Melinidinae, and Panicineae. Mya, million years ago; Pli, Pliocene; Plei, Pleistocene. Results from divergence time estimation based only on external angiosperm fossils calibration are shown in Fig 2. (PDF)
(PDF)

S3 Fig Ancestral range estimation (ARE) on the Panicinae chronogram using the BayArea+J model in BioGeoBEARS. Pie charts at nodes show the relative probability of the possible states (areas or combination of areas) before the instantaneous speciation event, whereas those on branches represent probability of the descendant lineage immediately after speciation. Boxes to the left of taxon names indicate areas of tip species. (PDF)
(PDF)
