# Supplementary material for: Molecular phylogeny of Panicum s. str. (Poaceae, Panicoideae, Paniceae) and insights into its biogeography and evolution
Source: PLoS One. 2018 Feb 21;13(2):e0191529. doi: 10.1371/journal.pone.0191529 (PMC5842878; doi:10.1371/journal.pone.0191529)
Supplement: S1 Appendix — New sequences are denoted by * and the voucher information is given (DOCX). (DOCX) [file pone.0191529.s001.docx]

**S1 Appendix. List of taxa of the molecular analysis and GenBank accession numbers**. New sequences are denoted by * and the voucher information is given.

**Tribe Andropogoneae.** *Cymbopogon flexuosus* (Nees ex Steud.) Will.Watson, AF117404; *Phacelurus digitatus* (Sibth. & Sm.) Griseb., AF117418*; Zea mays* L., U21985.

**Tribe Paniceae.** *Acroceras zizanioides* (Kunth) Dandy, AY029618; *Adenochloa* *adenophora* (Pilg.) Zuloaga, AY188454; *A.* *claytonii* (Renvoize) Zuloaga, AY188462; *Cenchrus ciliaris* L., AY029625; *C. setaceus* (Forssk.) Morrone, AY029673; *Dichanthelium acuminatum* (Sw.) Gould & C.A. Clark, AY188485; *Echinochloa colona* (L.) Link, AY029631; *Eriochloa punctata* (L.) Desv., AY029634; *Kellochloa brachyantha* (Steud.) Lizarazu, Nicola & Scataglini, KR232096; *K. verrucosa* (Muhl.) Lizarazu, Nicola & Scataglini, AY188496; *Lasiacis sorghoidea* (Desv.) Hitchc. & Chase, AY029639; *Louisiella elephantipes* (Nees ex Trin.) Zuloaga, AY029647, KF938890; *L. fluitans* C.E. Hubb. & J. Léonard, JN604693, KF938889; *Megathyrsus maximus* (Jacq.) B. K. Simon & S. W. L. Jacobs, AY029649; *Melinis repens* (Willd.) Zizka, AY029675; *Moorochloa eruciformis* (Sm.) Veldkamp, AY188452; *Oplismenus hirtellus* (L.) P.Beauv., AY029644; ***Panicum* s. str.:** *P. afzelii* Sw., MG581746* *Bille 3305*; *P. alatum* Zuloaga & Morrone, MG581747* *Morrone 3637*; *P. amarum* Elliott, HE577874, MG581748* *Zuloaga 15359*; *P.* *anabaptistum* Steud., MG581749* *Barbier 93*; *P. aquaticum* Poir., AY029658, KP878967; *P*. *aristispiculum* (B.K. Simon) Zuloaga, MG581750* *Forster 41372*; *P. australiense* Domin, KF938888, MG581743* *Forster 37563*; *P. bergii* Arechav., AY188457; *P. brassianum* (B.K. Simon) Zuloaga, MG581751* *Kahler 828*; *P. buncei* F. Muell. ex Benth., MG581752* *Eddie 1135*; *P. callosum* Hochst., MG581753* *Letouzey 7033*; *P. capillarioides* Vasey, MG581754* *Zuloaga 9687*; *P. carteri* Hosaka, MG581755* *Hosaka 2611*; *P. cervicatum* Chase, AY188459, KP878968; *P. chillagoanum* B.K. Simon, MG581756* *Wannan 5072*; *P. chloroleucum* Griseb., AY188460; *P. cinctum* Hack., LN908143; *P. clarksonianum* (B.K. Simon) Zuloaga, MG581757* *McDonald 2968*; *P. coloratum* Walter, AM849208, MG581758* *Poilecot 7805*; *P. curviflorum* Swallen, MG581759* *Sorensen 4439*; *P. deciduum* Swallen, MG581760* *Steyermark & Bunting* *103118*; *P. decompositum* R.Br., MG581761* *Morat 7363*; *P. deschampsioides* Domin, MG581762* *Hoober s*/*n*; *P. dichotomiflorum* Michx., AY188466; *P. fauriei* Hitchc., AY029650; *P. fluviicola* Steud., FR667672, MG581763* *Poilecot 2650*; *P. ghiesbreghtii* E. Fourn., MG581764* *Morrone 3631*; *P. griffonii* Franch., MG581765* *Dorsstach 136*; *P. hirticaule* J. Presl, MG581766* *Morrone 3628*; *P. impeditum* Launert, MG581767* *Smook 10689*; *P. laetum* Kunth, AM849175, MG581768* *Lisowski 1004*, MG581769* *Lisowski 197*; *P. laevinode* Lindl., MG581770* *McDonald 9522*; *P. larcomianum* Hughes, HE573520; *P. latzii* R.D. Webster, MG581771* *McDonald 8883*; *P. ligulare* Nees, MG581772* *Zuloaga 9031*; *P. majusculum* F. Muell. ex Benth., MG581745* *Wilson 143*; *P. miliaceum* L., AY188472, FR667670; *P.* *mystasipum* Zuloaga & Morrone, AY188474; *P. mitchelii* Benth., MG581773* *Forster 39665*; *P. muelleri* (Hughes) Lazarides, MG581744* *Thompson 3177;* *P. nephelophilum* Gaudich., AY029645; *P. nigerense* Hitchc., MG581774* *Fons 2231*; *P. olyroides* Kunth, AY188475; *P. paludosum* Hochst. ex A. Rich., HE586110; *P. pampinosum* Hitchc. & Chase, MG581775* *Morrone 3650*; *P. pansum* Rendle, FR667674, MG581776* *Gautier 372*; *P. parcum* Hitchc. & Chase, MG581777* *Morrone 3642*, MG581778* *Zuloaga 7382*; *P. paucinode* Stapf, MG581779* *McCallum &Webster 308A*; *P. perangustatum* Renvoize, MG581780* *Robinson 4293*; *P. phragmitoides* Stapf, FR667676, MG581781* *Gautier 2138*; *P. pilgeri* Mez, MG581782* *Letouzey 7683*; *P. pilgerianum* (Schweick.) Clayton, MG581783* *Seydel 2211*; *P. pinifolium* Chiov., MG581784* *Frozier 1015*; *P. psilopodium* Trin., MG581785* *Clemens* *17660*; *P. queenslandicum* Domin, MG581786* *Maiden 1899*; *P. racemosum* (P. Beauv.) Spreng., AY188481; *P. repens* L., AY029651; *P*. *robustum* B.K. Simon, MG581787* *Forster 36709*; *P. rudgei* Roem. & Schult., AY029661; *P. ruspolii* Chiov., MG581788* *Friis 2657*; *P.* *schinzii* Hack. ex Schinz, MG581789* *Poilecot 7786*; *P. seminudum* Domin, HE573555; *P. stramineum* Hitchc. & Chase, AY188489; *P. subalbidum* Kunth, HE575804; MG581790* *Friis 49*; *P. sumatrense* Roth, HE577875; *P. tricholaenoides* Steud., AY188493; *P. turgidum* Forssk., FR667675, MG581791* *Charpin 24684*; *P. urvilleanum* Kunth, MG581792* *Zuloaga 14978*; *P. venosum* Swallen GU594622; *P. virgatum* L., U21986. *P. voeltzkovii* A. Camus, LN908149, MG581793* *Humbert 29446*; **“*Panicum”* incertae sedis:** *P. acrotrichum* Hook. f., MG581800* *Carvallo 3757*; *P. aequinerve* Nees KR232095; *P. andringitrense* A. Camus, MG581801* *Humbert 6408*; *P. antidotale* Retz., AY188456; *P. bartletii* Swallen, JF804882; *P. bisulcatum* Thunb., FR821363, FR667671; *P. brevifolium* L. JF804860; *P. calvum* Stapf, MG581802* *Gereau 4722*; *P. capuronii* A. Camus, MG581803* *Humbert 29717*; *P. chionachne* Mez, KR232097; *P. comorense* Mez, LT593944, MG581804* *Kayombo 1209*; *P. eickii* Mez, KR232098; *P. gardneri* Thwaites, MG581805* *Clayton 1970*; *P. glandulopaniculatum* Renvoize, MG581806* *Jackson 1756*; *P.* *heterostachyum* Hack., JF804862; *P. hirtum* Lam, JF804861; *P. inaequilatum* Stapf, KR232099; *P.* *issongense* Pilg., MG581807* *Thompson 1006*; *P. laticomun* Nees, MG581808* *Bogdan 4715*; *P. millegrana* Poir., AY029660; *P. monticola* Hook. f., MG581809* *Robinson 5309*; *P. notatum* Retz., MG581810* *Larsen 8469*; *P. perrieri* A. Camus, LN908148; *P. pleianthum* Peter, HF558466; *P. pusillum* Hook. f., MG581811* *Philips 1518*; *P. pygmaeum* R. Br., HE573539; *P. robynsii* A. Camus, MG581812* *Poulsen 902*; *P. sellowii* Nees, AY188484; *P. subhystrix* A. Camus, LN908152; *P. trichanthum* Nees, AY188492, JF804858, HE573506; *P. trichocladum* Hack. ex K. Schum., MG581813* *Grenway 15368*; *P. trichoides* Sw. JF804859; *Parodiophyllochloa cordovensis* (E.Fourn.) Zuloaga & Morrone, AY188463; *Paspalidium geminatum* (Forssk.) Stapf, AY029662; *Pseudoechinolaena polystachya* (Kunth) Stapf, AY029676; *Sacciolepis* *africana* C.E. Hubb. & Snowden, FR821365; *S. angustissima* (Hochst. ex Steud.) Kuhlm., JF804856; *S. chevallieri* Stapf, HE573511; *S. indica* (L.) Chase, AY029677; *S. myuros* (Lam.) Chase, JF804857; *S. vilvoides* (Trin.) Chase, JF804854; *Setaria lachnea* (Nees) Kunth, AY029683; *S. viridis* (L.) P. Beauv., U21976; *Stenotaphrum secundatum (*Walter) Kuntze, AY029684; *Trichanthecium arctum* (Swallen) Zuloaga & Morrone, JF804881; *T. auricomum* (Nees ex Trin.) Zuloaga & Morrone, JF804870; *T. brazzavillense* (Franch.) Zuloaga & Morrone, JF804880; *T. caaguazuense* (Henrard) Zuloaga & Morrone, JF804875; *T. cyanescens* (Nees ex Trin.) Zuloaga & Morrone, JF804867; *T. dinklagei* (Mez) Zuloaga & Morrone, JF804866; T. *distichophyllum* (Spreng.) Zuloaga & Morrone, JF804869; *T. gracilicaule* (Rendle) Zuloaga & Morrone, JF804864; *T. granuliferum* (Kunth) Zuloaga & Morrone, JF804871; *T. machrisianum* (Swallen) Zuloaga & Morrone, JF804874; *T. margaritiferum* (Chiov.) Zuloaga & Morrone, JF804863; *T. micranthum* (Kunth) Zuloaga & Morrone, JF804877; *T. natalense* (Hochst.)Zuloaga & Morrone, JF804879; *T. nervosum* (Lam.) Zuloaga & Morrone, JF804876; *T. parvifolium* (Lam.) Zuloaga & Morrone, AY188476; *T. polycomum* (Trin.) Zuloaga & Morrone, JF804873; *T. pseudisachne* (Mez) Zuloaga & Morrone, JF804868; *T. pyrularium* (Hitchc. & Chase) Zuloaga & Morrone, JF804878; *T. rivale* (Swallen) Zuloaga & Morrone, JF804872; *T. schwackeanum* (Mez) Zuloaga & Morrone, AY188483; *T. tenellum* (Lam.) Zuloaga & Morrone, JF804865; *T. wettsteinii* (Hack.) Zuloaga & Morrone, AY188497; *Urochloa plantaginea* (Link) D. Webster, AY029693; *Whiteochloa airoides* (R. Br.) Lazarides, MG581794* *Roberts 4023*; *W. biciliata* Lazarides, MG581799* *Mitchel & Craig 7934*; *W. capillipes* (Benth.) Lazarides, JN604714, MG581795* *Adams 3046*; *W. cymbiformis* (Hughes) B.K. Simon, MG581797* *Glober 20*; *W. multiciliata* Lazarides, MG581798* *Lazarides 100*; *W. semitonsa* (F. Muell. ex Benth.) C.E. Hubb., MG581796* *Mitchel 15642*; *Zuloagaea* *bulbosa (*Kunth) Bess, AY029648.

**Tribe Paspaleae.** *Altoparadisium chapadense* Filg. et al., AY029619; *Anthaenantia lanata* (Kunth) Benth., AY029640; *Arthropogon villosus* Nees, AY029622; *Coleataenia prionitis* (Nees) Soreng, AY029652; *C. tenera* (Beyr. ex Trin.) Soreng, AY188491; *Cyphonanthus* *discrepans* (Döll) Morrone & Zuloaga, DQ646392; *Echinolaena inflexa* (Poir.) Chase, AY029633; *Homolepis glutinosa* (Sw.) Zuloaga & Soderstr., AY029637; *Hopia obtusa* (Kunth) Zuloaga & Morrone, AY029659; *Hymenachne donacifolia* (Raddi) Chase, AY029635; *H. pernambucensis* (Spreng.) Zuloaga, AY188478; *Mesosetum chaseae* Luces, AY029641; *Ocellochloa chapadensis* (Swallen) Zuloaga & Morrone, AY188486; *Oplismenopsis najada* (Hack. & Arechav.) Parodi, AY188453; *Otachyrium versicolor* (Döll) Henrard, AY029643; *Paspalum conjugatum* P.J. Bergius, AY029669; *Phanopyrum gymnocarpon (*Elliott) Nash, AY188469; *Plagiantha tenella* Renvoize,

AY029674; *Renvoizea sacciolepoides* (Renvoize & Zuloaga) Zuloaga & Morrone, EU107786; *R. trinii* (Kunth) Zuloaga &Morrone, EU107781; *Rugoloa hylaeica* (Mez) Zuloaga, AY188470; *Steinchisma hians* (Elliot) Nash, AY029685; *S. laxa* (Sw.) Zuloaga, AY029655; *Streptostachys* *asperifolia* Desv., AY029687.

**Tribe Thysanolaenae**. *Thysanolaena maxima* (Roxb.) Kuntze, U21984.

**Tribe Zeugiteae**. *Zeugites pittieri* Hack., U21987.

|  |
| --- |
