# Supplementary material for: Molecular phylogeny of Panicum s. str. (Poaceae, Panicoideae, Paniceae) and insights into its biogeography and evolution
Source: PLoS One. 2018 Feb 21;13(2):e0191529. doi: 10.1371/journal.pone.0191529 (PMC5842878; doi:10.1371/journal.pone.0191529)
Supplement: S1 Table — Area names in rows and columns are: A, North America; B Central and South America; C, Eurasia + Mediterran + North Africa; D, Tropical and South Africa; E, Southern Asia; F, Australia. For dispersal events the ancestral areas (where the lineage dispersed from) are given in the row, and the descendent areas (where the lineage dispersed to) are given in the column. (DOCX) (DOCX) [file pone.0191529.s002.docx]

**Table S1.** Areas and dispersal probabilities used in BioGeoBEARS analyses of subtribe Panicinae. Area names in rows and columns are: **A**, North America; **B** Central and South America; **C**, Eurasia + Mediterran + North Africa; **D**, Tropical and South Africa; **E**, Southern Asia; **F**, Australia. For dispersal events the ancestral areas (where the lineage dispersed from) are given in the row, and the descendent areas (where the lineage dispersed to) are given in the column.

|  | **A** | **B** | **C** | **D** | **E** | **F** |
| --- | --- | --- | --- | --- | --- | --- |
| **A** | 1 | 1 | 1 | 0.1 | 0.1 | 0.1 |
| **B** | 1 | 1 | 0.1 | 0.5 | 0.1 | 0.1 |
| **C** | 1 | 0.1 | 1 | 1 | 1 | 0.1 |
| **D** | 0.1 | 0.5 | 1 | 1 | 1 | 0.5 |
| **E** | 0.1 | 0.1 | 1 | 1 | 1 | 1 |
| **F** | 0.1 | 0.1 | 0.1 | 0.5 | 1 | 1 |
