# Supplementary material for: Molecular phylogeny of Panicum s. str. (Poaceae, Panicoideae, Paniceae) and insights into its biogeography and evolution
Source: PLoS One. 2018 Feb 21;13(2):e0191529. doi: 10.1371/journal.pone.0191529 (PMC5842878; doi:10.1371/journal.pone.0191529)
Supplement: S2 Table — Event counts were averaged across 1000 BSMs and are presented here with the standard deviations in parentheses. Total event counts are given for range-expansion events (anagenetic dispersal) (a), founder events (cladogenetic dispersal) (b), and Sympatry speciation events (c). For dispersal events the ancestral areas (where the lineage dispersed from) are given in the row, and the descendent areas (where the lineage dispersed to) are given in the column. The percentages of events involving each area either as a source (the rows) or as the destination (the columns), are given on the margins. Area names in rows and columns are: A, North America; B Central and South America; C, Eurasia + Mediterran + North Africa; D, Tropical and South Africa; E, Southern Asia; F, Australia. (DOCX) (DOCX) [file pone.0191529.s003.docx]

**Table S2.** Number of biogeographical events estimated in the history of the subtribe Panicinae using biogeographical stochastic mapping. Event counts were averaged across 1000 BSMs and are presented here with the standard deviations in parentheses. Total event counts are given for range-expansion events (anagenetic dispersal) (a), founder events (cladogenetic dispersal) (b), and Sympatry speciation events (c). For dispersal events the ancestral areas (where the lineage dispersed from) are given in the row, and the descendent areas (where the lineage dispersed to) are given in the column. The percentages of events involving each area either as a source (the rows) or as the destination (the columns), are given on the margins. Area names in rows and columns are: **A**, North America; **B** Central and South America; **C**, Eurasia + Mediterran + North Africa; **D**, Tropical and South Africa; **E**, Southern Asia; **F**, Australia.

| **(a)** Range-expansion event counts (anagenetic dispersal), mean and (standard deviations). | | | | | | | |
| --- | --- | --- | --- | --- | --- | --- | --- |
| **Area** | **A** | **B** | **C** | **D** | **E** | **F** | **%** |
| **A** | - | 0.66 (0.67) | 0 (0) | 0 (0) | 0 (0) | 0 (0) | **6.60** |
| **B** | 2.34 (0.67) | - | 0 (0) | 0 (0) | 0 (0) | 0 (0) | **23.39** |
| **C** | 0 (0) | 0 (0) | - | 0.09 (0.29) | 0 (0) | 0 (0) | **0.93** |
| **D** | 0 (0) | 0 (0) | 0.91 (0.29) | - | 1.20 (0.80) | 0.72 (0.65) | **28.29** |
| **E** | 0 (0) | 0 (0) | 0 (0) | 0.90 (0.77) | - | 0.76 (0.75) | **16.60** |
| **F** | 0 (0) | 0 (0) | 0 (0) | 0.98 (0.74) | 1.44 (0.81) | - | **24.19** |
| **%** | **23.39** | **6.60** | **9.10** | **19.72** | **26.39** | **14.80** |  |

| **(b)** Founder event counts (cladogenetic dispersal), mean and (standard deviations). | | | | | | | |
| --- | --- | --- | --- | --- | --- | --- | --- |
| **Area** | **A** | **B** | **C** | **D** | **E** | **F** | **%** |
| **A** | - | 0.21 (0.54) | 0.024 (0.15) | 0.18 (0.4) | 0.08 (0.27) | 0.01 (0.071) | **2.32** |
| **B** | 1.3 (0.59) | - | 0.98 (0.17) | 4.34 (1.97) | 1.04 (0.6) | 1.34 (0.71) | **42.18** |
| **C** | 0.01 (0.063) | 0.04 (0.19) | - | 0.01 (0.15) | 0 (0) | 0.01 (0.089) | **0.30** |
| **D** | 0.91 (0.48) | 4.35 (1.85) | 0.087 (0.28) | - | 1.68 (0.73) | 0.65 (0.75) | **35.98** |
| **E** | 0.39 (0.49) | 0.58 (0.65) | 0 (0) | 0.54 (0.66) | - | 0.09 (0.29) | **7.49** |
| **F** | 0.01 (0.055) | 0.21 (0.41) | 1 (0) | 1.12 (0.35) | 0.17 (0.39) | - | **11.73** |
| **%** | **26.06** | **53.87** | **20.90** | **61.90** | **29.65** | **20.90** |  |

| **(c)** Sympatry speciation event count, mean and (standard deviations). | | | | | | |
| --- | --- | --- | --- | --- | --- | --- |
| **Area** | **A->A,A** | **AB->AB,AB** | **B->B,B** | **D->D,D** | **E->E,E** | **F->F,F** |
| **Mean (SD)** | 3.89 (0.27) | 7.59 (0.03) | 23.15 (1.97) | 31.51 (1.97) | 2.66 (0.51) | 31.19 (0.68) |
| **%** | **3.89** | **7.59** | **23.15** | **31.51** | **2.66** | **31.19** |
