# Supplementary figures and images for: Molecular phylogeny of Panicum s. str. (Poaceae, Panicoideae, Paniceae) and insights into its biogeography and evolution
Source: PLoS One. 2018 Feb 21;13(2):e0191529. doi: 10.1371/journal.pone.0191529 (PMC5842878; doi:10.1371/journal.pone.0191529)

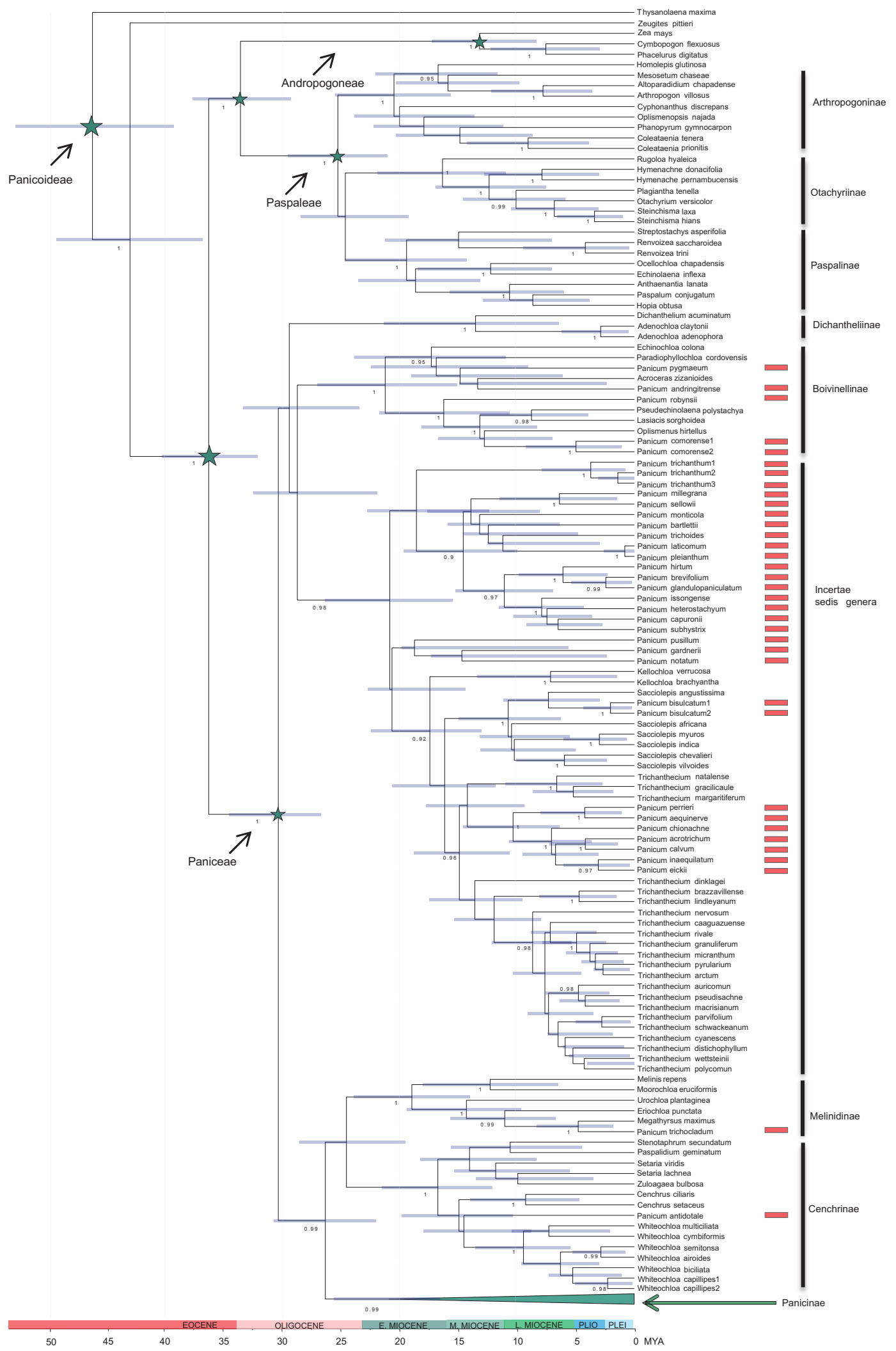

Supplement: S1 Fig — Red boxes indicate phylogenetic placement of Panicum species recovered outside subtribe Panicinae. Posterior probability ≥ 0.9 are shown on the branches and horizontal bars on the nodes indicate the 95% HPD of ages. Subtribe Panicinae are shown in detail in S2 Fig Mya, million years ago; Pli, Pliocene; Plei, Pleistocene. Results from divergence time estimation based only on external angiosperm fossils calibration are shown in Fig 1. (PDF) (PDF) [file pone.0191529.s004.pdf]

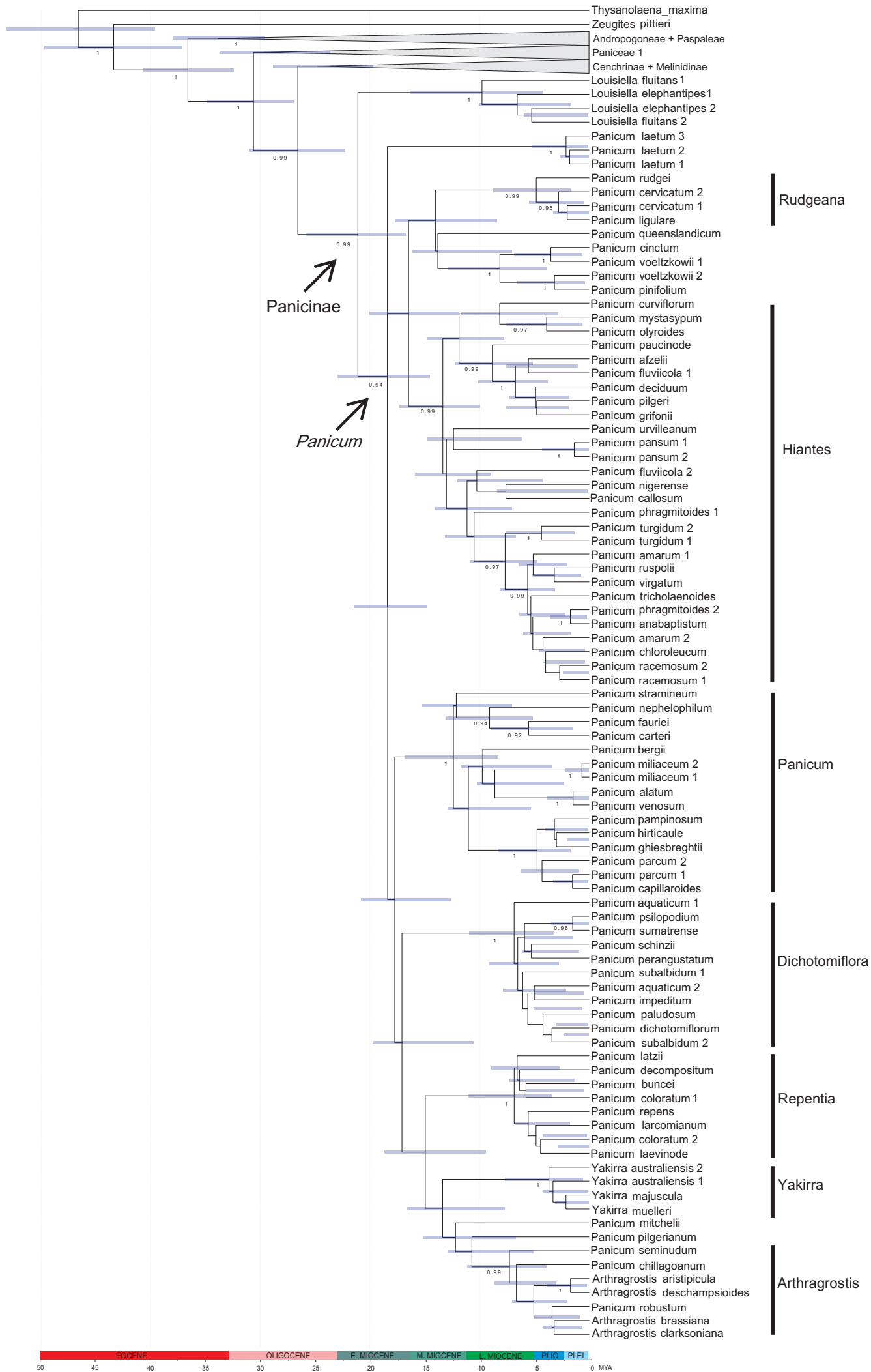

Supplement: S2 Fig — Maximum clade credibility (MCC) tree of Panicoideae obtained from BEAST analyses with ndhF sequences using the uncorrelated lognormal relaxed clock model and secondary calibrations based on external angiosperm fossils together with the phytolith microfossils of Poaceae (calibration scheme 2, see materials and methods). Only subtribe Paniceae is shown in detail; for the remaining clades see S1 Fig Posterior probabilities ≥ 0.9 are shown on the branches and horizontal bars on the nodes indicate the 95% HPD of ages. Vertical bars indicate sections within Panicum. Paniceae 1 refers to tribe Paniceae excluding subtribes Cenchrinae, Melinidinae, and Panicineae. Mya, million years ago; Pli, Pliocene; Plei, Pleistocene. Results from divergence time estimation based only on external angiosperm fossils calibration are shown in Fig 2. (PDF) (PDF) [file pone.0191529.s005.pdf]

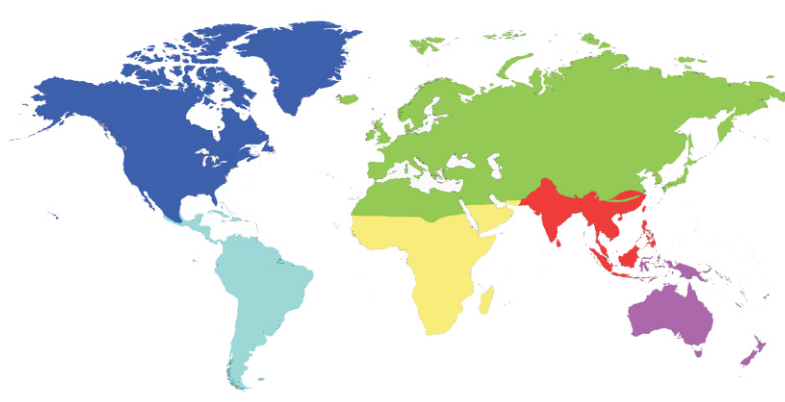

- A** North America
- B** Neotropics
- C** Eurasia
- D** Sub-Saharan Africa
- E** Southeast Asia
- F** Australia

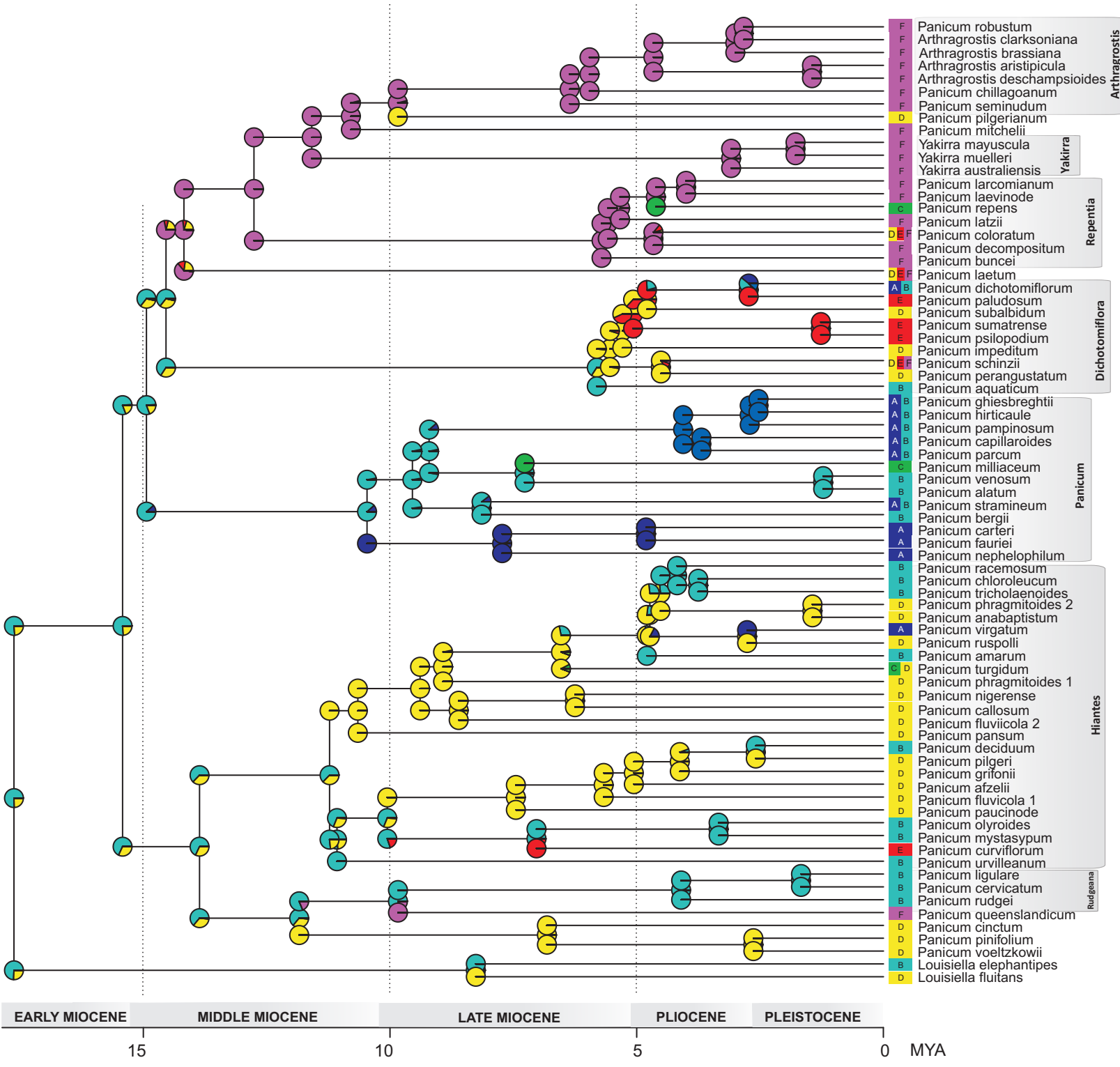

Supplement: S3 Fig — Pie charts at nodes show the relative probability of the possible states (areas or combination of areas) before the instantaneous speciation event, whereas those on branches represent probability of the descendant lineage immediately after speciation. Boxes to the left of taxon names indicate areas of tip species. (PDF) (PDF) [file pone.0191529.s006.pdf]
